# Integrated Bulk and Single-Nucleus Transcriptomics Characterizes the Developmental and Regional Specialization of Chicken Abdominal Fat-Associated Tissue

**DOI:** 10.3390/ani16182893

**Published:** 2026-09-14

**Authors:** Siyu Zhang, Qihong Zhang, Jiali Wang, Zhenhui Li

**Affiliations:** 1 State Key Laboratory of Swine and Poultry Breeding Industry, Guangdong Provincial Key Lab of Agro-Animal Genomics and Molecular Breeding, College of Animal Science, South China Agricultural University, Guangzhou 510642, China; zhangsiyu@faas.cn (S.Z.);; 2 Fujian Key Laboratory of Animal Genetics and Breeding, Institute of Animal Husbandry and Veterinary Medicine, Fujian Academy of Agricultural Sciences, Fuzhou 350013, China; 3 Guangdong Laboratory for Lingnan Modern Agriculture, Guangzhou 510642, China

**Keywords:** chicken, adipose tissue, abdominal fat, anatomical specialization

## Abstract

Excessive abdominal fat deposition reduces feed efficiency and edible carcass yield in chickens, yet its developmental and regional specialization remains poorly understood. We generated 93 bulk RNA sequencing (RNA-seq) libraries from 15 fat-associated anatomical regions at embryonic day 16 (E16) and 16 regions at 2 weeks post-hatch (2w) in six Xinghua chickens. Exploratory single-nucleus RNA sequencing was additionally performed on abdominal region (AR) tissue from one individual at each stage. Repeated-measures-aware analysis identified developmental stage as the major source of transcriptomic variation, with 4556 genes expressed at higher levels at E16 and 4528 at 2w. E16-associated programs were enriched for proliferation, morphogenesis, and structural development, whereas postnatal programs were dominated by immune and stimulus-response processes. Stage-specific weighted gene co-expression network analysis (WGCNA) identified substantial regional heterogeneity. AR showed stage-dependent signatures, including higher developmental and morphogenetic programs and lower lipid-metabolic programs at E16, and higher cell-cycle-related and lower developmental and fatty-acid-metabolic programs at 2w. Single-nucleus analysis identified adipose-lineage and multiple non-adipocyte populations, and cross-omics mapping showed that AR-associated signatures were distributed across multiple cellular compartments. These results suggest stage-dependent, multicellular transcriptional specialization of chicken AR tissue.

## Simple Summary

Excessive abdominal fat reduces feed efficiency and edible carcass yield in chickens, but fat-associated tissues at different anatomical sites do not necessarily develop in the same way. We analyzed 93 tissue samples collected from 15 anatomical regions at embryonic day 16 and 16 regions at 2 weeks after hatching in six Xinghua chickens, together with exploratory single-nucleus RNA sequencing of the abdominal region. Developmental stage accounted for much of the gene-expression variation: embryonic tissues were enriched for genes related to tissue growth and structure, whereas postnatal tissues showed stronger immune- and environmental-response signals. Differences among anatomical regions were also evident within each stage. In the abdominal region, stage-dependent expression patterns were represented in several cell populations, including fat-related, stromal, epithelial, vascular, neural-associated, and immune cells. Our results show that abdominal fat development involves both adipose-related processes and the surrounding cellular environment.

## 1. Introduction

Excessive abdominal fat deposition is an undesirable trait in chicken production because it diverts dietary energy toward a depot that contributes little to edible meat yield. Genetic selection has markedly improved broiler growth and feed efficiency, but fat deposition remains a complex trait affected by genotype, developmental stage, nutrition, and tissue-specific metabolic regulation [1,2,3]. In commercial processing, abdominal fat is generally removed or discarded, and therefore excessive accumulation of this depot reduces carcass yield and feed-use efficiency [4]. Understanding why abdominal fat accumulates differently from other anatomically distinct fat-associated tissues is therefore relevant for both poultry breeding and the biological study of adipose tissue development.

Adipose tissue is not a uniform lipid-storage compartment. It is a metabolically active connective tissue composed of adipocytes, preadipocytes, vascular cells, immune cells, stromal cells, and other depot-associated cell populations [5,6]. In mammals, white adipose tissue is recognized as a dynamic and heterogeneous organ involved in energy homeostasis, lipid and glucose handling, vascular regulation, endocrine signaling, and host defense [7]. Single-cell and single-nucleus studies have revealed extensive heterogeneity among adipocytes, adipose progenitors, immune cells, and vascular cells, with marked differences between subcutaneous and visceral fat-associated tissues and across metabolic states [6,8,9]. These findings indicate that differences among fat-associated tissues are not only anatomical but also cellular and molecular.

In poultry, abdominal fat has been investigated extensively because of its economic importance, whereas adipose tissues at other anatomical sites remain comparatively underexplored. Fat-associated tissues occur near the skin, muscles, joints, neck, orbit, wings, legs, and thoracic structures and may represent subcutaneous or regionally specialized adipose tissues [10]. Transcriptomic studies have shown that chicken abdominal and subcutaneous adipose tissues differ in their developmental trajectories and in pathways related to lipid metabolism, extracellular matrix organization, immune activity, and adipocyte differentiation [11,12]. Treating all adipose tissues as equivalent may therefore obscure biologically important regional differences. However, it remains unclear how abdominal fat is transcriptionally distinguished from a broad range of anatomically distributed fat-associated tissues.

Developmental timing is another important dimension of adipose biology. In chickens, adipose tissue growth is shaped by both prenatal tissue patterning and postnatal nutritional and metabolic adaptation. Chicken adipose tissues undergo substantial transcriptional remodeling from embryonic development to the post-hatch period, including depot-dependent changes in adipocyte differentiation, lipid metabolism, extracellular matrix organization, and immune activity [4,12,13]. Previous studies have investigated abdominal fat deposition in chickens using candidate genes, quantitative trait loci, genome-wide association analyses, transcriptomics, and nutritional interventions [1,14,15]. These studies have provided valuable information on genes and pathways associated with fatness, lipid metabolism, and adipogenesis. However, many previous studies have focused on abdominal fat alone, comparisons between fat and lean lines, or a limited number of developmental stages and fat-associated tissues [4,11,12,16,17]. As a result, relatively little is known about how abdominal fat becomes transcriptionally distinct from anatomically distinct fat-associated tissues during early development.

Bulk RNA sequencing enables the comparison of gene expression across multiple adipose tissues and developmental stages, supporting the identification of differentially expressed genes, enriched pathways, and adipose depot-specific transcriptional signatures. Weighted gene co-expression network analysis (WGCNA) further organizes genes into co-expression modules and links these modules and candidate hub genes to traits such as anatomical site and developmental stage [18,19]. However, bulk RNA-seq measures average expression across heterogeneous cell populations and cannot identify the cellular origin of transcriptional signals [5,6]. Single-cell and single-nucleus RNA sequencing provide complementary cellular resolution, with single-nucleus RNA-seq being particularly suitable for adipose tissue because mature adipocytes are large, fragile, and difficult to capture by conventional single-cell dissociation [20,21]. Studies in human and mouse white adipose tissues have revealed depot-specific cellular heterogeneity and associations between specific cell populations and metabolic traits [5,6,22]. Although cell-resolved studies of chicken adipose tissue are emerging, its developmental and multi-depot cellular specialization remains poorly understood [23].

Xinghua chicken is a Chinese indigenous breed with important genetic and production value. Compared with highly selected commercial broilers, indigenous breeds may provide useful models for studying natural variation in growth, fat deposition, and tissue development. In the present study, we asked whether abdominal fat has distinct transcriptional and functional characteristics compared with other anatomically distinct fat-associated tissues, and how these characteristics change from embryonic to early postnatal development.

To address this question, we performed bulk RNA-seq on 93 samples representing 15 embryonic and 16 postnatal anatomically distinct fat-associated tissues, including abdominal and visceral fat-related tissues. We further generated single-nucleus RNA-seq profiles from abdominal fat at the embryonic and postnatal stages. By integrating differential expression analysis, functional enrichment, stage-specific WGCNA, single-nucleus cell-type annotation, and bidirectional bulk–single-nucleus mapping, we aimed to identify developmental and tissue-specific gene programs associated with abdominal fat specialization.

## 2. Materials and Methods

### 2.1. Experimental Animals and Tissue Collection

Xinghua chickens were examined at embryonic day 16 (E16) and 2 weeks post-hatch (2w), representing prenatal and early postnatal development, respectively. Fertilized eggs were obtained from Fengkai County Zhicheng Poultry Breeding Co., Ltd. (Zhaoqing, Guangdong Province, China) and incubated at 38 °C and approximately 60% relative humidity under controlled hatchery conditions. Eggs were automatically turned every 2 h until embryonic day 19 (E19), after which turning was discontinued until hatching. After hatching, chicks were housed at five birds per 1.5 m^2^ cage (approximately 3.3 birds/m^2^) at approximately 28 °C, under ambient relative humidity and a natural photoperiod. Water and a commercial starter diet containing approximately 20% crude protein and 11.91 MJ/kg metabolizable energy were provided ad libitum.

**Figure 1:**
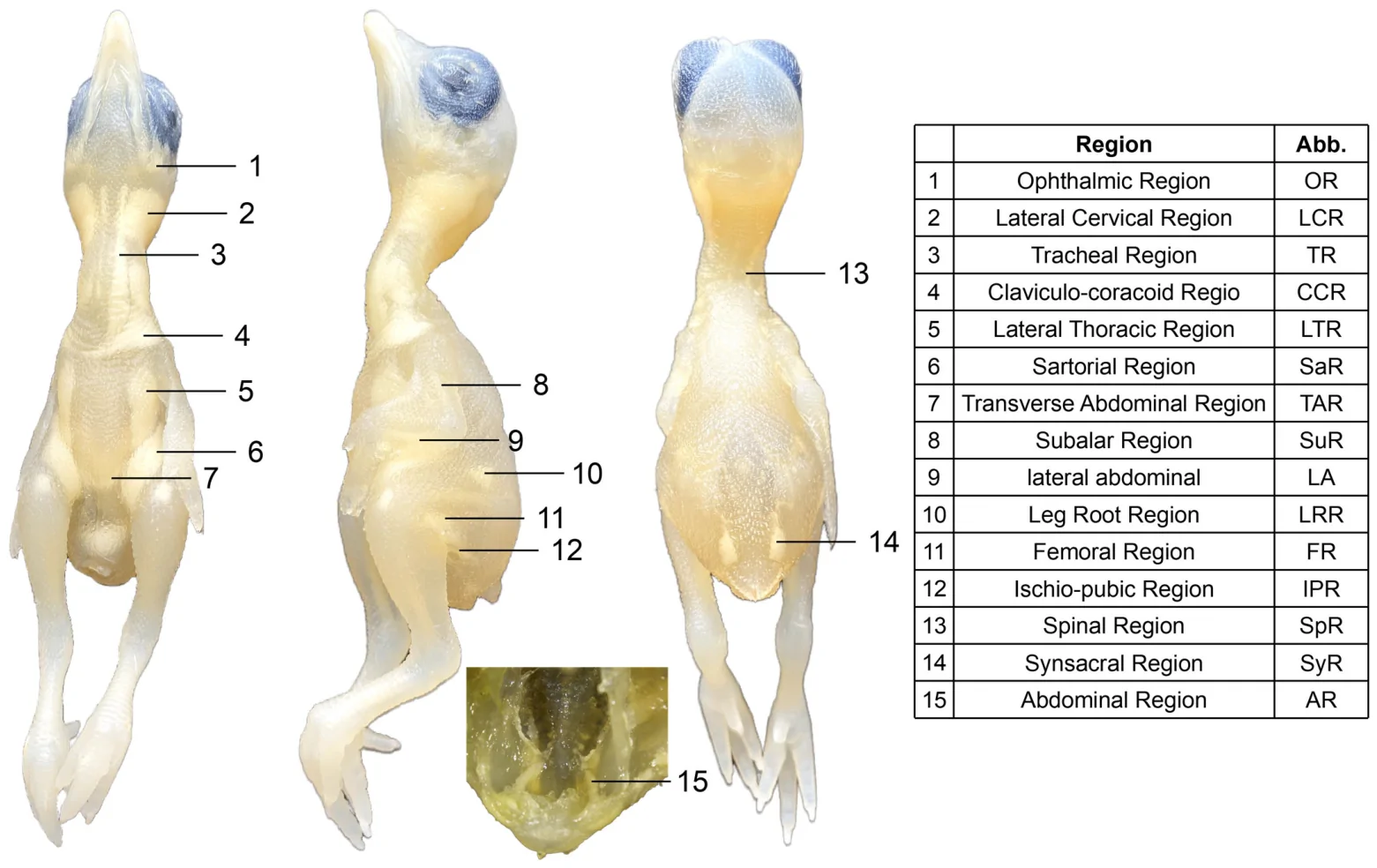
Anatomical distribution of fat-associated regions sampled from an embryonic Xinghua chicken. Representative whole-mount images of an embryonic day 16 (E16) Xinghua chicken after CUBIC-L optical clearing are shown in ventral, lateral, and dorsal views. The numbered labels indicate the 15 anatomically defined fat-associated regions sampled at E16: 1, OR; 2, LCR; 3, TR; 4, CCR; 5, LTR; 6, SaR; 7, TAR; 8, SuR; 9, LA; 10, LRR; 11, FR; 12, IPR; 13, SpR; 14, SyR; and 15, AR. Full anatomical names, sampling landmarks, and operational classifications are provided in Table 1. The inset shows an enlarged view of AR. VR is not shown because a discrete visceral depot could not be reproducibly separated at E16 and was collected only at 2 weeks post-hatch.

Three biological individuals were sampled at each developmental stage. Only females were included in the study. At E16, sex was determined by gonadal morphology, with females identified by asymmetric gonadal development. At 2w, sex was initially assessed based on comb morphology and subsequently confirmed by ovarian examination at dissection. Fifteen fat-associated anatomical regions were collected from each E16 female embryo, whereas the same 15 regions plus an additional visceral fat region (VR) were collected from each 2w female chick (Figure 1, Table 1). This within-individual multi-region design generated 93 tissue transcriptomes (45 at E16 and 48 at 2w) from six biological individuals. VR was collected only at 2w because a discrete perigastric visceral fat region could not be reproducibly separated at E16.

Tissues were processed immediately or snap-frozen in liquid nitrogen and stored at −80 °C until RNA extraction or nuclei isolation. All procedures were approved by the Institutional Animal Care and Use Committee of South China Agricultural University (approval no. SCAU#2024A027).

### 2.2. Whole-Embryo Optical Clearing

Whole E16 embryos were cleared using a modified CUBIC-L protocol [24]. Embryos were immersed in a solution containing 10% (*w*/*w*) *N*-butyldiethanolamine (N802391, MACKLIN, Shanghai, China) and 10% (*w*/*w*) Triton X-100 (T6328, MACKLIN) and incubated at 37 °C with gentle agitation for 10 days. The clearing solution was replaced three times during incubation. Cleared embryos were used to document the anatomical locations of the sampled anatomically distinct fat-associated tissues.

### 2.3. Bulk RNA Sequencing and Data Processing

Poly(A)-enriched, strand-specific mRNA libraries were prepared using BGI library preparation reagents according to the manufacturer’s instructions and sequenced on the DNBSEQ-T7 platform to generate 150 bp paired-end reads. Sample-level sequencing depth and alignment statistics are provided in Table S1.

Raw reads were filtered and trimmed using fastp (v0.23.2) [25] and aligned to the Gallus GRCg7b reference genome using STAR (v2.7.11a) [26]. Gene-level counts were generated using --quantMode GeneCounts, and genes with fewer than 10 total counts in the relevant dataset were excluded.

Raw gene-count matrices were used for downstream differential-expression analyses as described below. Variance-stabilizing transformation was performed using DESeq2 (v1.50.2) [27], and the resulting expression matrix was used for descriptive principal component analysis, sample-correlation analysis, expression visualization, and WGCNA.

### 2.4. Differential Expression and Functional Enrichment Analyses

For the global developmental comparison, analyses were restricted to the 15 anatomical regions shared between E16 and 2w. Lowly expressed genes were filtered using edgeR, followed by TMM normalization and voomWithDreamWeights transformation. Repeated measurements from the same individuals were accounted for using dream with the model ~ Stage + Region + (1 | Individual), with Kenward–Roger small-sample correction. Robustness was further assessed using the original DESeq2 fixed-effects model (~Stage + Region) and an individual-level sensitivity analysis. For the latter, voom-normalized log-expression values were equally averaged across the 15 shared regions within each animal, yielding six independent individual-level profiles (three E16 and three 2w) [28]. These profiles were analyzed using limma with a stage-only linear model (~Stage), followed by empirical-Bayes moderation. Concordance among the dream, DESeq2, and individual-level analyses was quantified using pairwise Spearman correlations of gene-level log2 fold-change estimates and the proportion of genes with concordant effect directions.

Developmental differences were also analyzed separately within each of the 15 shared regions using DESeq2 (~Stage). AR-associated expression patterns were evaluated separately at each stage using individual identity as a blocking factor (~Individual + Region). AR was compared with the equally weighted mean of the other 14 regions at E16. At 2w, the primary comparison excluded VR, whereas sensitivity analyses included VR in the reference group and directly compared AR with VR.

Genes with a Benjamini–Hochberg FDR < 0.05 were considered statistically significant. For downstream analyses requiring more stringent regional signatures, a high-confidence subset was defined by FDR < 0.05 and |log2 fold change| > 2. Functional enrichment analyses were performed using clusterProfiler [29], including Gene Ontology (GO) Biological Process.

### 2.5. Weighted Gene Co-Expression Network Analysis

WGCNA was performed using variance-stabilized bulk RNA-seq expression values [18]. Samples and genes were evaluated using goodSamplesGenes. Signed co-expression networks and signed topological overlap matrices were constructed using soft-thresholding powers selected according to scale-free topology fit. Modules were identified by hierarchical clustering and dynamic tree cutting with a minimum module size of 30 genes and were merged at a cut height of 0.25. Three networks were constructed: a global network using all 93 tissue transcriptomes, an E16-specific network using 45 transcriptomes from 15 regions, and a 2w-specific network using 48 transcriptomes from 16 regions. Global developmental-stage associations were tested across the 15 regions shared between stages using a mixed-effects model (ME ~ Stage + Region + (1 | Individual)). Within each developmental stage, module–region associations were evaluated using individual-blocked linear models (ME ~ Individual + Region), with each focal region contrasted against the equally weighted mean of the remaining regions. *p* values were adjusted using the Benjamini–Hochberg method. Modules with significant region associations (FDR < 0.05) were ranked by their maximum absolute t statistic, and representative modules were selected for visualization and functional interpretation. GO Biological Process enrichment was performed using clusterProfiler, with genes assigned to the corresponding stage-specific WGCNA network used as the background.

### 2.6. Single-Nucleus RNA Sequencing and Cell-Type Annotation

Single-nucleus RNA sequencing was performed on AR tissue from one E16 embryo and one 2w chick. These data were used for exploratory cell-type annotation and cross-omics mapping rather than population-level developmental inference.

Nuclei were isolated from frozen tissues by mechanical dissociation in precooled nuclei isolation buffer, followed by filtration and centrifugation. Nuclear concentration and integrity were assessed by acridine orange/propidium iodide staining. Libraries were generated using the BGI DNBelab C4 platform and sequenced on a BGI DNBSEQ-T7.

Expression matrices were analyzed using Seurat v5 [30]. Seurat objects were created with a minimum of 200 detected features per nucleus. Nuclei were retained when they met the following criteria: 200 < nFeature_RNA < 6000, 500 < nCount_RNA < 40,000, and mitochondrial transcript percentage < 10%. Doublets were detected independently in each sample using scDblFinder (v1.24.10) [31].

After quality control, the embryonic and postnatal datasets were analyzed independently. Each dataset was normalized and scaled, followed by highly variable gene selection, PCA, neighbor-graph construction, clustering, and UMAP visualization. Cell types were annotated separately for each stage based on canonical and cluster-enriched marker genes. The annotated datasets were then merged and integrated using Harmony (v2.0.5) [32] based on the first 25 principal components for joint visualization and comparison across stages.

### 2.7. Cross-Omics Integration

Bulk and single-nucleus RNA-seq datasets were integrated using complementary gene-set overlap, enrichment, and scoring approaches. First, the 2000 most highly expressed genes in preadipocytes and adipocytes at each developmental stage were intersected with stage-matched AR-upregulated and AR-downregulated bulk DEGs, generating eight gene sets defined by stage, cell type, and expression direction. These gene sets were subjected to functional enrichment analysis. Second, enrichment of single-nucleus cell-type marker genes within stage-specific bulk WGCNA modules was evaluated using hypergeometric tests. Marker and module genes were restricted to the corresponding WGCNA gene universe, and enrichment *p* values were adjusted globally using the Benjamini–Hochberg method. Overlap sizes, enrichment ratios, and intersecting genes were recorded. Third, high-confidence AR-associated bulk signatures (FDR < 0.05 and |log2FC| > 2) were scored across annotated cell populations in the stage-matched single-nucleus datasets to evaluate the cellular distribution of AR-associated transcriptional signatures. Finally, adipose-lineage marker-enriched WGCNA modules showing significant negative associations with AR in the individual-blocked analysis (FDR < 0.05) were selected for functional characterization. GO Biological Process enrichment was performed using clusterProfiler (v4.18.4), with all genes in the corresponding stage-specific WGCNA network used as the background. For all adipose-lineage marker-enriched WGCNA modules, intramodular connectivity (kWithin) was calculated, and the expression patterns of the top-ranked candidate hub genes were visualized across anatomical regions.

Gene identifiers were harmonized across datasets using org.Gg.eg.db. Ensembl version suffixes were removed, and Ensembl identifiers were converted to gene symbols or Entrez identifiers as required. Analysis-specific background gene sets were used for overlap and enrichment tests to account for differences in gene representation among datasets.

All analyses were conducted in R v4.5. Figures were generated using ggplot2 (v4.0.3), ComplexHeatmap (v2.26.1), pheatmap (v1.0.13), enrichplot (v1.30.5), and patchwork (v1.3.2).

## 3. Results

### 3.1. Developmental Stage Dominates Transcriptomic Variation Across Chicken Fat-Associated Tissues

**Figure 2:**
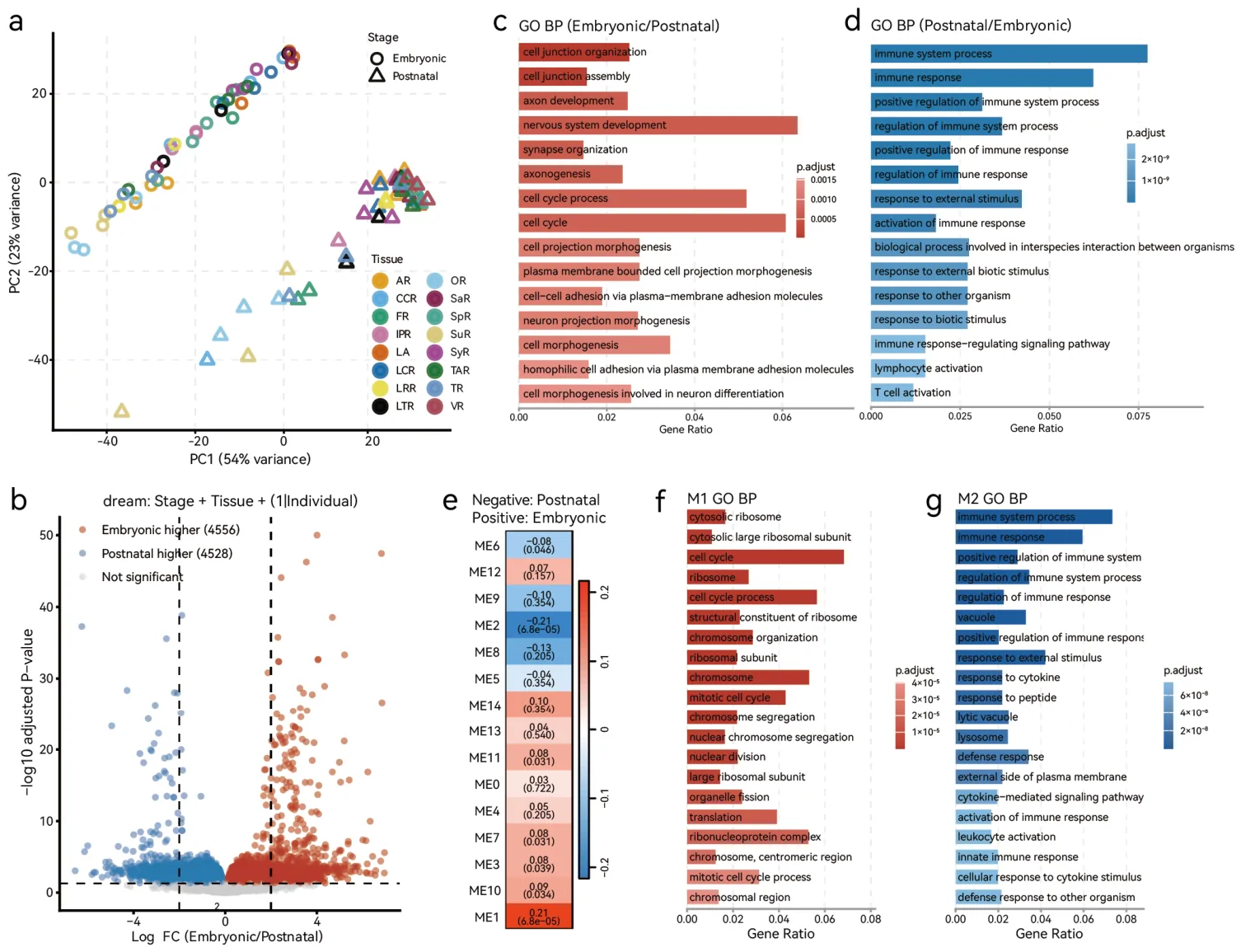
Global transcriptomic remodeling during the embryonic-to-postnatal transition of chicken fat-associated tissues. (**a**) Principal component analysis of all 93 bulk RNA-seq samples based on variance-stabilized expression values. Colors indicate anatomical regions, and shapes indicate developmental stages. (**b**) Volcano plot of the repeated-measures-aware developmental comparison across the 15 anatomical regions shared between E16 and 2w. Positive log2 fold-change values indicate higher expression at E16, and negative values indicate higher expression at 2w. Red and blue points indicate genes with FDR < 0.05 at E16 and 2w, respectively. (**c**,**d**) GO Biological Process enrichment of genes expressed at significantly higher levels at E16 and 2w, respectively. (**e**) Repeated-measures-adjusted developmental-stage effects on global WGCNA module eigengenes. Positive effect estimates indicate higher eigengene expression at E16, and negative estimates indicate higher expression at 2w. (**f**,**g**) GO biological process enrichment of representative E16- and 2w-associated module groups, respectively. Dot or bar color represents the adjusted *p* value, and gene ratio represents the proportion of input genes assigned to each term.

Bulk RNA-seq generated 93 tissue transcriptomes from six biological individuals, comprising 45 transcriptomes from 15 fat-associated anatomical regions at E16 and 48 transcriptomes from 16 regions at 2w. PCA of variance-stabilized expression values showed clear separation between the two developmental stages, with PC1 and PC2 accounting for 54% and 23% of the total variance, respectively (Figure 2a). Sample–sample correlation analysis further showed strong overall similarity among transcriptomes from the same developmental stage, while retaining region- and individual-associated variation (Figure S1). Consistent with these patterns, gene-wise variance partitioning indicated that developmental stage represented a substantial source of biological expression variation, together with contributions from anatomical region and individual identity (Figure S2). These results supported the presence of pronounced developmental differences while also highlighting the hierarchical and repeated-measures structure of the dataset.

To account for repeated sampling of multiple anatomical regions from the same individuals, the global developmental comparison was performed across the 15 regions shared between E16 and 2w using a repeated-measures-aware mixed-effects model. At FDR < 0.05, 4556 genes showed higher expression at E16 and 4528 showed higher expression at 2w (Figure 2b; Table S2). E16-higher genes were enriched for developmental and structural processes, including cell-cycle regulation, nervous system development, axonogenesis, cell adhesion, and cell morphogenesis (Figure 2c; Table S3). In contrast, genes expressed at higher levels at 2w were predominantly associated with immune-system processes, regulation and activation of immune responses, cytokine-related responses, and responses to external or biotic stimuli (Figure 2d; Table S3). Gene-level effect estimates were highly concordant across the three developmental analyses (Figure S3). Pairwise Spearman correlations of log2 fold-change estimates were *p* = 0.992 for dream versus DESeq2, *p* = 0.996 for dream versus the individual-level analysis, and ρ = 0.994 for DESeq2 versus the individual-level analysis. Relative to the primary dream analysis, effect directions agreed for 97.4% of genes in the DESeq2 analysis and 99.3% in the individual-level analysis; among genes with |dream log2FC| ≥ 0.5, directional concordance with the individual-level analysis increased to 99.9%. These results support the robustness of the broad developmental expression pattern across analytical frameworks.

Region-wise developmental comparisons across the 15 shared anatomical regions broadly recapitulated these patterns. Although the specific enriched terms varied among regions, E16-higher genes were generally associated with tissue development, cell proliferation, morphogenesis, and extracellular-matrix organization, whereas 2w-higher genes were more frequently associated with immune regulation, cytokine signaling, defense responses, and responses to external stimuli (Figures S4–S6).

Repeated-measures-aware analysis of the global WGCNA network further identified stage-associated co-expression programs (Figure 2e; Table S4). ME1 showed higher eigengene expression at E16 (β = 0.21, FDR = 6.8 × 10^−5^), whereas ME2 showed higher expression at 2w (β = −0.21, FDR = 6.8 × 10^−5^). ME1 was enriched for ribosomal, cell-cycle, chromosome-organization, and mitotic processes (Figure 2f; Table S5), whereas ME2 was enriched for immune-system processes, cytokine responses, lysosomal functions, and responses to external stimuli (Figure 2g; Table S6).

### 3.2. Stage-Specific Co-Expression Networks Characterize Regional Transcriptomic Variation

**Figure 3:**
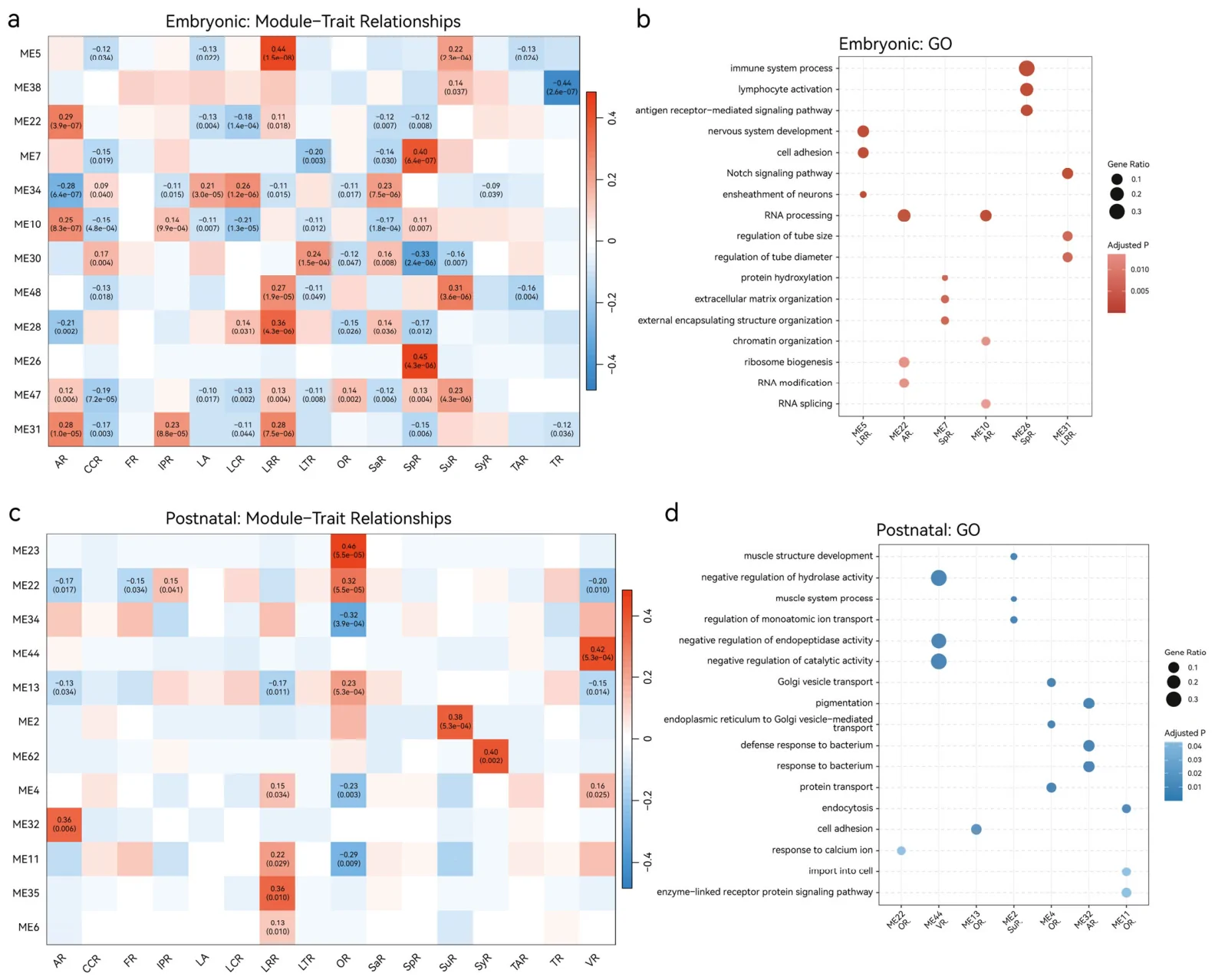
Stage-specific WGCNA identifies region-associated co-expression programs in chicken fat-associated tissues. (**a**) Module–region associations in the embryonic WGCNA network. Each tile shows the effect estimate from the individual-blocked linear model, with FDR values shown in parentheses. (**b**) GO Biological Process enrichment of representative embryonic module–region associations. Module names and their associated anatomical regions are indicated on the *x*-axis. Dot size represents the gene ratio, and dot color represents the adjusted *p* value. (**c**) Module–region associations in the postnatal WGCNA network, displayed as in (**a**). (**d**) GO Biological Process enrichment of representative postnatal module–region associations. Dot size represents the gene ratio, and dot color represents the adjusted *p* value.

Because developmental stage was a major source of transcriptomic variation, separate WGCNA networks were constructed for the embryonic and postnatal datasets to characterize region-associated co-expression patterns. Stage-specific PCA further illustrated substantial within-stage regional variation, with several anatomical regions occupying distinct positions in the PCA space (Figure S7). Individual-blocked analyses of module eigengenes identified multiple modules showing positive or negative associations with specific anatomical regions at both developmental stages (Figure 3a,c; Tables S7 and S9).

At the embryonic stage, several representative modules showed pronounced regional associations. ME5 was positively associated with LRR (effect estimate = 0.44, FDR = 1.51 × 10^−8^), whereas ME7 and ME26 were positively associated with SpR (effect estimates = 0.40 and 0.45, respectively). AR was positively associated with ME22 and ME10 (effect estimates = 0.28 and 0.25, FDR = 6.36 × 10^−7^ and 4.33 × 10^−6^, respectively), while ME31 showed a positive association with LRR (effect estimate = 0.28) (Figure 3a). Functional enrichment of these representative module–region pairs suggested distinct biological programs across anatomical regions (Figure 3b; Table S8). The LRR-associated ME5 was enriched for nervous system development, cell adhesion, and ensheathment of neurons, whereas ME31 was associated with Notch signaling and regulation of tube size and diameter. The SpR-associated ME7 was enriched for extracellular matrix and external encapsulating structure organization, while ME26 was associated with immune-system processes, lymphocyte activation, and antigen receptor-mediated signaling. Notably, the two AR-associated modules showed enrichment for RNA-related processes: ME22 was associated with RNA processing, ribosome biogenesis, and RNA modification, whereas ME10 was associated with RNA processing, chromatin organization, and RNA splicing.

Region-associated co-expression patterns were also evident in the postnatal network (Figure 3c). ME32 was positively associated with AR (effect estimate = 0.36, FDR = 5.78 × 10^−3^), whereas ME44 showed a positive association with the postnatal-specific visceral region VR (effect estimate = 0.42, FDR = 5.27 × 10^−4^). ME2 was positively associated with SuR (effect estimate = 0.38, FDR = 5.27 × 10^−4^). Several modules were also associated with OR, including positive associations for ME22 (effect estimate = 0.32, FDR = 5.53 × 10^−5^) and ME13 (effect estimate = 0.23, FDR = 5.27 × 10^−4^), and negative associations for ME4 (effect estimate = −0.23, FDR = 3.01 × 10^−3^) and ME11 (effect estimate = −0.29, FDR = 9.01 × 10^−3^). Functional enrichment further distinguished these regional programs (Figure 3d; Table S10). The VR-associated ME44 was enriched for negative regulation of hydrolase, endopeptidase, and catalytic activities, whereas the AR-associated ME32 was enriched for pigmentation and responses to bacteria. The SuR-associated ME2 was linked to muscle structure development, muscle-system processes, and regulation of monoatomic ion transport. OR-associated modules encompassed several additional processes, including calcium-ion responses, cell adhesion, Golgi and endoplasmic-reticulum-to-Golgi vesicle transport, endocytosis, and enzyme-linked receptor protein signaling. The region-associated modules differed markedly in functional composition between the embryonic and postnatal networks.

### 3.3. Single-Nucleus RNA-Seq Characterizes Cell Populations in the Abdominal Region

**Figure 4:**
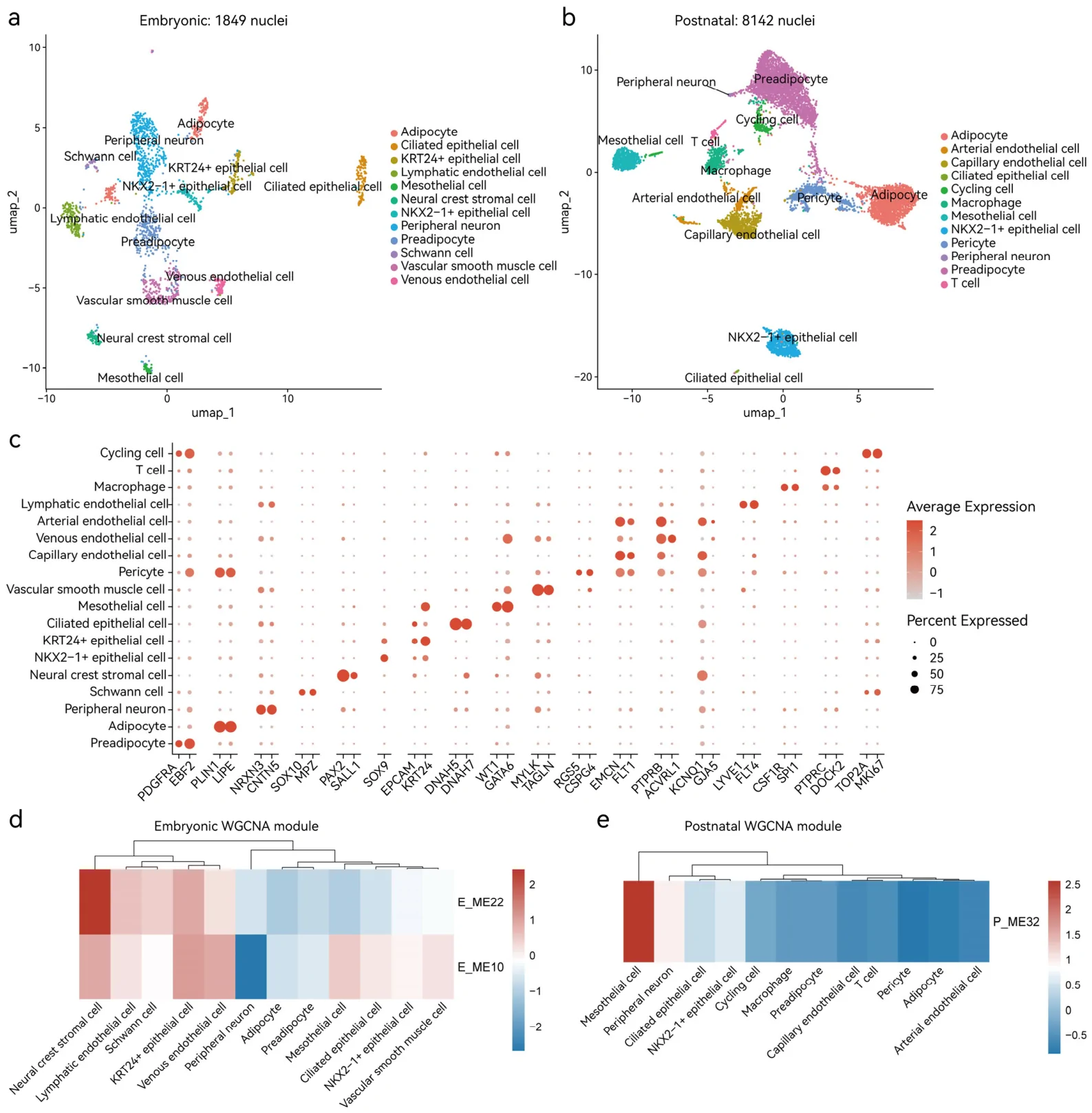
Single-nucleus transcriptomic atlas of embryonic and postnatal abdominal fat-associated tissue. (**a**,**b**) Unintegrated UMAP visualizations of 1849 embryonic and 8142 postnatal nuclei. (**c**) Expression of representative marker genes across annotated cell populations. Dot size represents the percentage of nuclei expressing each gene, and color represents scaled average expression. (**d**,**e**) Cell-type-level mean module scores of representative AR-associated WGCNA modules.

Exploratory single-nucleus RNA-seq was performed on AR tissue from one E16 embryo and one 2w chick. Stage-specific analyses were first performed independently to visualize the cellular structure of each sample without cross-sample integration (Figure 4a,b; Table S11). After quality control and doublet removal, 1849 embryonic and 8142 postnatal nuclei were retained. The two datasets were subsequently integrated to facilitate joint cell-type annotation and visualization, with the combined and stage-split embeddings shown in Figure S9. Because only one biological individual was analyzed at each stage, the integrated data were used for annotation and transcriptomic contextualization rather than for population-level comparisons of cell abundance or developmental differences.

The embryonic AR sample contained annotated preadipocytes and adipocytes together with peripheral neurons, Schwann cells, lymphatic and venous endothelial cells, vascular smooth muscle cells, mesothelial cells, neural crest stromal cells, NKX2-1+ epithelial cells, KRT24^+^ epithelial cells, and ciliated epithelial cells (Figure 4a). The postnatal AR sample likewise contained preadipocytes and adipocytes, together with capillary and arterial endothelial cells, pericytes, macrophages, T cells, cycling cells, peripheral neurons, mesothelial cells, NKX2-1^+^ epithelial cells, and ciliated epithelial cells (Figure 4b). These annotations illustrate the cellular complexity of the sampled AR tissue but should not be interpreted as quantitative differences in cellular composition between developmental stages.

Cell-type annotations were supported by representative marker genes (Figure 4c; Tables S12 and S13). Preadipocytes expressed stromal and adipogenic-progenitor markers including PDGFRA and EBF2, whereas adipocytes expressed *PLIN1* and *LIPE*. Endothelial and mural populations were supported by markers including *FLT1*, *MCAM*, *RGS5*, *MYLK*, and *TAGLN*, whereas immune and cycling populations expressed markers such as *SPI1*, *PTPRC*, *TOP2A*, and *MKI67*.

Projection of representative AR-associated WGCNA modules onto the stage-matched single-nucleus datasets suggested that the corresponding bulk co-expression programs were distributed across multiple cellular compartments (Figure 4d,e). In the embryonic sample, E_ME22 showed its highest relative module score in neural crest stromal cells, with comparatively high scores also observed in several endothelial, Schwann, and epithelial populations. E_ME10 showed relatively high scores in neural crest stromal and KRT24^+^ epithelial cells, together with positive scores in several mesothelial and epithelial populations, whereas its score was comparatively low in peripheral neurons (Figure 4d). In the postnatal sample, P_ME32 showed a markedly higher relative score in mesothelial cells than in the other annotated populations, with lower scores across most adipose-lineage, vascular, immune, and epithelial populations (Figure 4e). These exploratory mappings suggest that AR-associated bulk co-expression patterns are not restricted to adipocytes or preadipocytes and may reflect contributions from stromal, epithelial, mesothelial, neural-associated, and vascular compartments. However, module scores do not establish the cellular origin or regulatory function of these bulk transcriptional signals.

### 3.4. Stage-Dependent AR-Associated Bulk Signatures and Their Cellular Context

**Figure 5:**
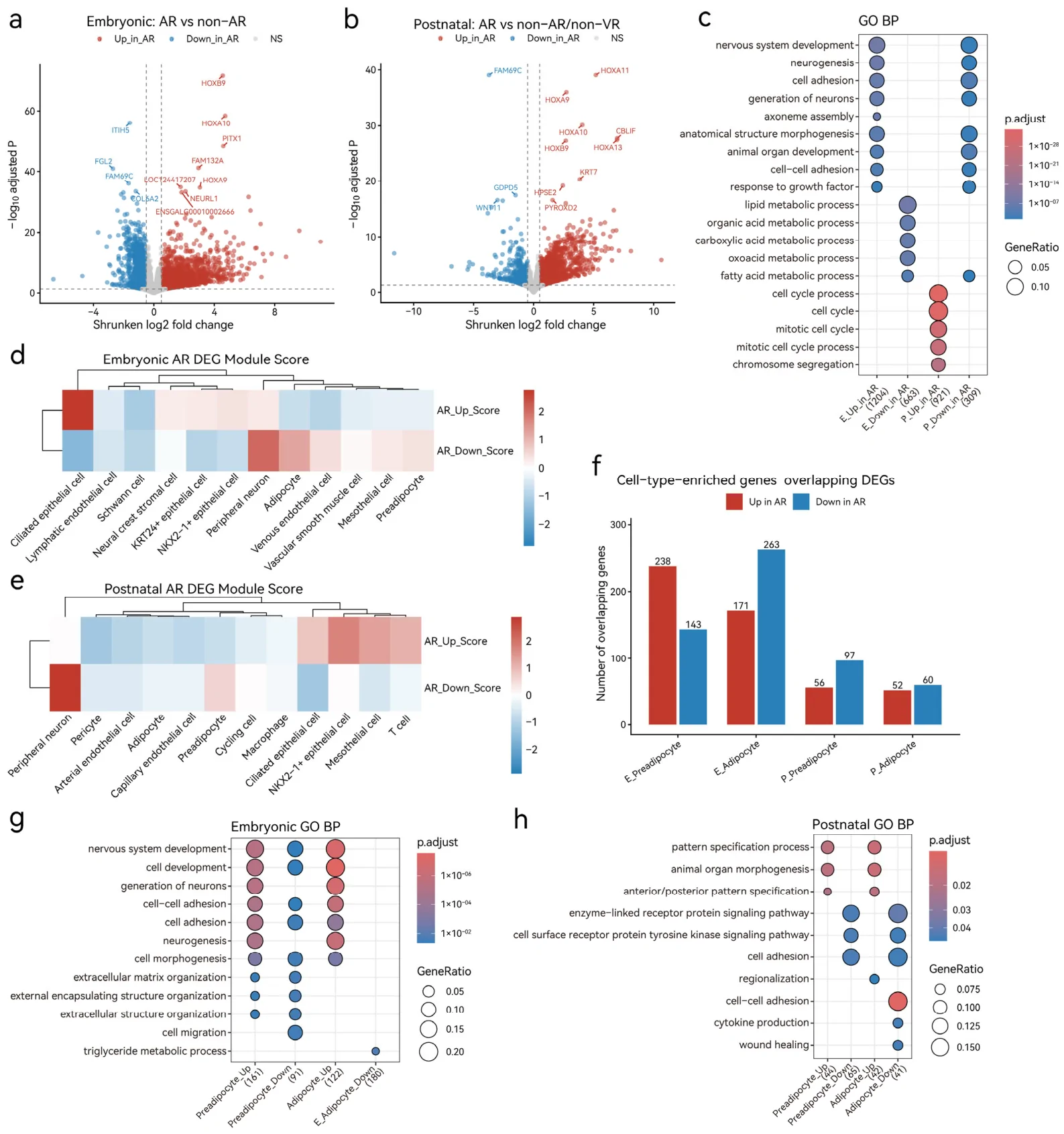
Stage-dependent AR-associated transcriptional signatures and their cell-type and functional associations. (**a**,**b**) Volcano plots of individual-blocked comparisons between AR and the equally weighted mean of the reference regions at E16 and 2w, respectively. Positive log2 fold-change values indicate higher expression in AR. (**c**) GO biological process enrichment of genes expressed at higher or lower levels in embryonic and postnatal AR. (**d**,**e**) Mean module scores of high-confidence AR-upregulated and AR-downregulated signatures across embryonic and postnatal cell populations, respectively. (**f**) Numbers of AR-associated DEGs overlapping with the 2000 most highly expressed genes in preadipocytes and adipocytes. Gene overlap represents shared gene sets and does not imply causal regulation. (**g**,**h**) GO biological process enrichment of the overlapping gene sets at the embryonic and postnatal stages, respectively. Numbers in parentheses indicate the numbers of input genes. Dot size represents gene ratio, and dot color represents the adjusted *p* value.

Stage-specific, individual-blocked analyses were used to characterize AR-associated expression patterns. At E16, AR was compared with the equally weighted mean of the other 14 regions, whereas at 2w, the primary reference excluded VR. These comparisons identified 2177 AR-higher and 1230 AR-lower genes at E16, and 1420 AR-higher and 560 AR-lower genes at 2w (Figure 5a,b; Tables S14 and S15). Because VR was an additional visceral-type region available only at 2w, we further assessed its influence on the postnatal AR signature. Direct AR–VR comparison identified 1549 AR-higher and 717 VR-higher genes, while inclusion of VR in the reference group produced highly concordant AR effect estimates (Spearman *r* = 0.998; Figure S8a,b). AR-higher genes in the direct AR–VR comparison were enriched for cell-cycle and chromosome-segregation processes, whereas no significant GO Biological Process terms were identified among VR-higher genes (Figure S8c). These analyses suggest that the major postnatal AR-associated pattern was robust to the treatment of VR.

Functional enrichment of the primary AR comparisons showed clear stage-dependent differences (Figure 5c; Table S16). Embryonic AR-higher genes were associated mainly with nervous-system development, neurogenesis, cell adhesion, morphogenesis, and growth-factor responses, whereas AR-lower genes were enriched for lipid- and fatty-acid-related metabolic processes. At 2w, AR-higher genes were predominantly associated with cell-cycle and chromosome-segregation processes, whereas AR-lower genes were associated with neural development, cell adhesion, morphogenesis, and fatty-acid metabolism.

High-confidence AR-associated signatures (FDR < 0.05 and |log2 fold change| > 2) were then mapped to the stage-matched single-nucleus datasets. In the embryonic sample, the AR-higher signature was most prominent in ciliated epithelial cells, whereas the AR-lower signature was expressed across peripheral neurons, adipocytes, endothelial cells, mesothelial cells, and preadipocytes. In the postnatal sample, the AR-higher signature showed relatively high scores in epithelial, mesothelial, and T-cell populations, whereas the AR-lower signature was most prominent in peripheral neurons (Figure 5d,e). These patterns suggest that AR-associated bulk signatures involve multiple cellular compartments rather than adipose-lineage cells alone.

Finally, AR-associated DEGs were intersected with the 2000 most highly expressed genes in preadipocytes and adipocytes (Figure 5f). Functional enrichment of these overlapping gene sets highlighted neural development, cell adhesion, extracellular-matrix organization, and triglyceride metabolism at E16, and pattern specification, receptor signaling, adhesion, cytokine production, and wound healing at 2w (Figure 5g,h; Tables S17 and S18). These overlaps represent shared gene sets and should be interpreted as exploratory cellular contextualization rather than evidence of causal regulation.

### 3.5. Adipose-Lineage Marker Enrichment Identifies WGCNA Modules Associated with AR

**Figure 6:**
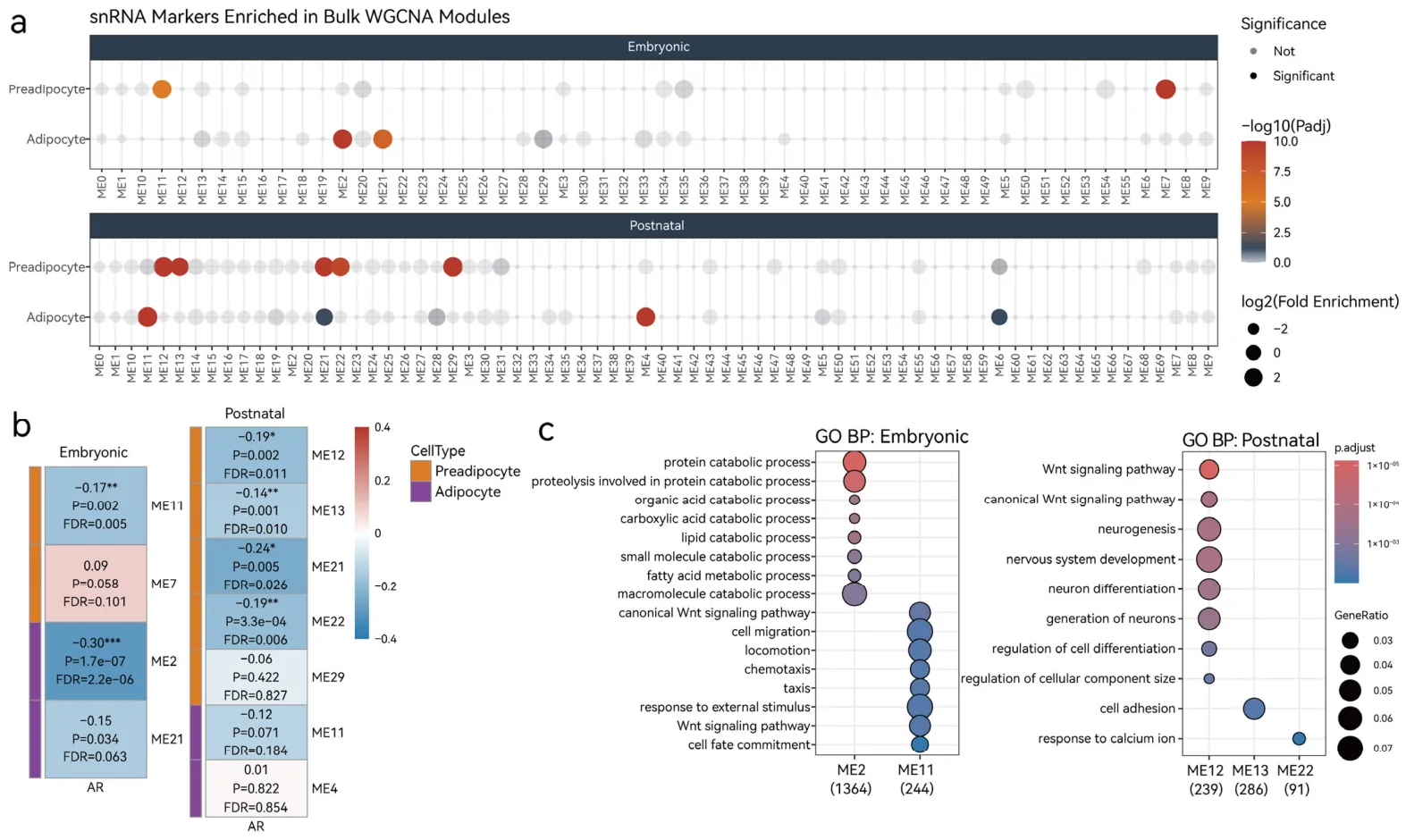
Single-nucleus marker enrichment within bulk WGCNA modules identifies adipose-lineage modules associated with AR. (**a**) Hypergeometric enrichment of preadipocyte and adipocyte marker genes in embryonic and postnatal WGCNA modules. Dot color represents −log10 adjusted *p* value, dot size represents log2 fold enrichment, and black outlines indicate statistically significant enrichment. (**b**) Associations between adipose-lineage marker-enriched modules and AR. Row annotations indicate the associated cell population. Each tile shows the effect estimate, nominal *p* value, and FDR. Asterisks indicate significance after Benjamini-Hochberg correction: * FDR < 0.05, ** FDR < 0.01, and *** FDR < 0.001. (**c**) GO biological process enrichment of embryonic ME2, ME11 and postnatal ME12, ME13 and ME22. Dot size represents gene ratio, and dot color represents the adjusted *p* value.

To relate stage-specific bulk co-expression modules to adipose-lineage cell populations, preadipocyte and adipocyte marker genes identified from the single-nucleus datasets were tested for enrichment within the corresponding WGCNA modules using background-controlled hypergeometric tests (Figure 6a). In the embryonic network, preadipocyte marker enrichment was observed in ME11 and ME7, whereas adipocyte marker enrichment was observed in ME2 and ME21. In the postnatal network, preadipocyte markers were enriched in ME12, ME13, ME21, ME22, and ME29, whereas adipocyte markers were enriched in ME11 and ME4.

Among these marker-enriched modules, several showed significant negative associations with AR (Figure 6b). At the embryonic stage, preadipocyte-enriched ME11 was negatively associated with AR, and adipocyte-enriched ME2 showed a stronger negative association. In contrast, the AR associations of embryonic ME7 and ME21 did not remain significant after multiple-testing correction. At the postnatal stage, four of the five preadipocyte-enriched modules showed significant negative associations with AR: ME12, ME13, ME21, and ME22. In contrast, ME29 and the adipocyte-enriched modules ME11 and ME4 showed no significant association with AR after FDR correction. These results identify several adipose-lineage-marker-enriched co-expression programs whose eigengene expression was relatively lower in AR than in other sampled regions. Consistent with the module-level results, the top candidate hub genes of adipose-lineage marker-enriched modules generally showed relatively lower expression in AR than in other anatomical regions, particularly for modules negatively associated with AR (Figure S10).

Functional enrichment further distinguished the biological characteristics of these modules (Figure 6c; Table S19). Embryonic ME2 was enriched for protein, organic-acid, carboxylic-acid, lipid, small-molecule, fatty-acid, and macromolecule catabolic processes. Embryonic ME11 was associated with canonical Wnt signaling, cell migration, locomotion, chemotaxis, responses to external stimuli, and cell-fate commitment. Among the postnatal modules, ME12 was enriched for canonical Wnt signaling, neurogenesis, nervous-system development, neuronal differentiation, and regulation of cell differentiation; ME13 was primarily associated with cell adhesion, and ME22 was associated with responses to calcium ions. No significant GO Biological Process term was identified for P_ME21 under the applied enrichment criteria. These marker-enrichment and module–region associations provide candidate co-expression programs linked to AR specialization but do not establish pathway activity or regulatory effects on adipogenesis.

## 4. Discussion

Across the 15–16 anatomical regions examined, developmental stage accounted for a major component of transcriptomic variation, but considerable regional heterogeneity remained within each stage. AR-associated bulk signatures and co-expression modules were represented in both adipose-lineage and non-adipocyte populations. This combination of developmental and regional variation suggests that early chicken fat-associated tissues are influenced by both developmental state and local cellular composition. Interpretation of the single-nucleus results remains exploratory because only one individual was analyzed at each stage, and the sampled tissues were anatomically, rather than histologically, defined.

Previous chicken transcriptomic studies have generally focused on abdominal fat, high- versus low-fat phenotypes, or comparisons among a small number of major adipose depots. Previous studies have characterized chicken subcutaneous adipose tissue across embryonic and post-hatch development [11,13], and clavicular fat across post-hatch development [12]. WGCNA studies of chickens divergent for abdominal-fat percentage have further identified PPAR-, fatty-acid-metabolism-, and MAPK-related modules associated with fatness [33]. In contrast, our 15-region embryonic and 16-region postnatal design shows that regional heterogeneity extends well beyond the conventional abdominal-versus-subcutaneous comparison. Stage-specific modules involved extracellular-matrix, immune, neural-associated, RNA-processing, chromatin, muscle-associated, transport, and tissue-response programs. Such diversity is biologically plausible because fat-associated tissues contain not only adipocytes but also stromal, vascular, immune, neural, epithelial, and connective-tissue components. Recent chicken single-cell studies likewise identified tissue-associated adipocyte and stromal heterogeneity between abdominal fat and other anatomical contexts [23]. Therefore, some regional signals detected by bulk RNA-seq probably reflect both cell-intrinsic transcriptional differences and variation in local cellular composition. The direct AR–VR comparison further showed that the two visceral fat-associated regions were transcriptionally distinguishable. AR-higher genes were enriched for cell-cycle and chromosome-related processes, while inclusion of VR in the reference set did not materially alter the overall AR-associated signature. This supports the robustness of the AR pattern to reference-region selection (Figure S8; Tables S15 and S16).

The single-nucleus mapping further supports this multicellular interpretation. Embryonic AR-associated modules E_ME22 and E_ME10 showed relatively high scores in neural crest stromal cells and were also represented across epithelial, endothelial, Schwann, mesothelial, and other populations rather than being concentrated in adipocytes or preadipocytes. Their bulk functional annotations included RNA processing, ribosome biogenesis, chromatin organization, and RNA splicing. These findings are compatible with active developmental and structural programs within the embryonic AR microenvironment. Developmental studies in mammals have shown that neural crest-derived cells can contribute to subsets of adipose stromal progenitors, although this contribution varies among depots and species [34]. In chickens, recent multi-omic and 3D-genomic analyses have also demonstrated extensive chromatin and regulatory remodeling associated with abdominal-fat deposition [35]. Nevertheless, the present module scores cannot establish lineage origin or regulatory function. A similar caution applies to neural-associated signatures: adipose innervation is increasingly recognized as an important component of adipose physiology, including sensory-neural regulation involving PIEZO2 [36], but neural signals in our AR samples may also originate from peripheral innervation or neighboring neural-associated tissue rather than adipocytes themselves.

The postnatal P_ME32 module was most prominent in mesothelial cells, providing further evidence that AR-associated bulk programs are not restricted to the adipose lineage. Mesothelial and mesothelial-like stromal populations are increasingly recognized as components of visceral adipose niches. An omentum-specific IGFBP2-expressing mesothelial-like stromal population has been reported to inhibit adipose-progenitor differentiation [37], whereas lineage-tracing studies in mice caution against interpreting mesothelial identity as direct evidence of adipocyte origin [38]. Accordingly, the strong P_ME32 score in chicken mesothelial cells is better interpreted as evidence that an AR-associated co-expression program is represented within a visceral stromal compartment.

Marker-guided WGCNA further identified adipose-lineage-enriched modules with relatively lower eigengene expression in AR. These modules involved lipid and organic-acid catabolism at the embryonic stage and Wnt-, adhesion-, differentiation-, and calcium-responsive processes postnatally. Wnt signaling is particularly relevant because studies of human adipose progenitors have identified Wnt-responsive structural cell states that retain progenitor features, while adipogenic cells differentiate along a parallel trajectory. Related developmental pathways, including Wnt, NOTCH, PPAR, and TGF-β signaling, have also been implicated in chicken adipogenesis, and functional studies such as NUMB–NOTCH1 manipulation provide experimental evidence that these pathways can influence chicken preadipocyte proliferation and differentiation [39]. These results extend previous WGCNA and multi-omic studies of chicken abdominal-fat deposition [33,35], by linking several co-expression programs to anatomical variation.

Several limitations define the scope of these conclusions. Although 93 bulk libraries were analyzed, they originated from only six biological individuals, with multiple regions sampled from each animal. Repeated-measures-aware and individual-blocked models were therefore used, but the limited number of individuals restricts population-level inference. Only E16 and 2w were examined, preventing reconstruction of a continuous developmental trajectory across the peri-hatch period and later growth. In addition, the single-nucleus datasets contained one individual per stage, so developmental and individual effects cannot be separated, and cell-abundance differences cannot be statistically inferred. Finally, anatomical sampling inevitably included non-adipocyte components, and histological validation was not available for every region. Future studies integrating replicated single-nucleus or spatial transcriptomics with histology, additional developmental stages, quantitative depot phenotypes, genetic variation, and functional perturbation will be required to distinguish cell-compositional effects from cell-intrinsic regulation and to determine which candidate programs contribute directly to abdominal-fat deposition.

## 5. Conclusions

This study identified developmental and regional transcriptomic differences among chicken fat-associated tissues. The abdominal region showed stage-dependent expression patterns involving both adipose-lineage and non-adipocyte populations, and WGCNA identified several co-expression programs associated with regional variation. Further biological replication and functional studies are needed to determine the roles of these candidate programs in abdominal fat deposition.

## Figures and Tables

**Table 1:** Anatomical definitions and operational classifications of the fat-associated regions sampled in this study.

Abbreviation	Full Name	Anatomical Location/Sampling Landmark	Operational Classification	Stage
OR	Ophthalmic region	Between the external acoustic meatus and the angle of the jaw, with a thin extension toward the eye	Subcutaneous-type	E16, 2w
LCR	Lateral cervical region	Lateral cervical region of the neck	Subcutaneous-type	E16, 2w
TR	Tracheal region	Along the lateral side of the trachea, extending toward the claviculo-coracoid region	Subcutaneous-type	E16, 2w
CCR	Claviculo-coracoid region	At the cervicothoracic junction where the neck joins the body, extending from anterior to the wing base toward the mid-ventral line	Subcutaneous-type	E16, 2w
LTR	Lateral thoracic region	On the superficial pectoral muscle between the leg and wing	Subcutaneous-type; muscle-associated	E16, 2w
SaR	Sartorius region	On the superficial surface of the sartorius muscle, following its outline	Subcutaneous-type; muscle-associated	E16, 2w
TAR	Transverse abdominal region	Along the posterior margin of the superficial pectoral muscle, extending from the sternal crest toward the subalar region	Subcutaneous-type; muscle-associated	E16, 2w
SuR	Subalar region	Immediately posterior to the base of the wing	Subcutaneous-type	E16, 2w
LA	Lateral abdominal region	In the inguinal region along the lateral abdominal wall	Subcutaneous-type	E16, 2w
LRR	Leg root region	Proximal hindlimb/leg-root region according to the sampling landmark used in this study	Subcutaneous-type	E16, 2w
FR	Femoral region	Overlying the semitendinosus and semimembranosus muscles of the thigh	Subcutaneous-type; muscle-associated	E16, 2w
IPR	Ischiopubic region	Immediately anterior and dorsal to the cloacal opening, overlying the posterior portions of the ischium and pubis	Subcutaneous-type	E16, 2w
SpR	Spinal region	Dorsal spinal region extending posteriorly toward the level of the subalar region and associated with the anterior spinal feather tract	Subcutaneous-type	E16, 2w
SyR	Synsacral region	Following the contour of the synsacrum in the dorsal pelvic region	Subcutaneous-type	E16, 2w
AR	Abdominal region	Intra-abdominal fat region; enlarged in Figure 1 inset	Visceral-type	E16, 2w
VR	Visceral fat region	Intra-abdominal fat surrounding the proventriculus and gizzard	Visceral-type	2w only

Note: Anatomical nomenclature for most embryonic regions was adapted from Liebelt and Eastlick [10], with additional study-specific regions defined by gross anatomical landmarks. Tissue classifications are operational and based on sampling location rather than histological composition. “Muscle-associated” denotes a subcutaneous-type region located directly on or adjacent to skeletal muscle and does not indicate a separately defined perimuscular adipose depot. VR refers to perigastric visceral fat surrounding the proventriculus and gizzard. E16, embryonic day 16; 2w, 2 weeks post-hatch.

## Data Availability

The raw sequencing data, processed bulk/snRNA matrices and sample/cell metadata generated in this study have been deposited in the China National Nucleotide Sequence Archive (CNSA) under project accession number CNP0009931.

## Supplementary Materials

The following supporting information can be downloaded at: https://www.mdpi.com/article/10.3390/ani16182893/s1, Figure S1: Sample–sample correlation structure of the 93 bulk RNA-seq tissue transcriptomes; Figure S2: Gene-wise variance partitioning of bulk RNA-seq expression across developmental stage, anatomical region, biological individual, and residual variation; Figure S3: Robustness of the global embryonic-to-postnatal transcriptomic comparison across alternative analytical frameworks; Figure S4: Region-specific transcriptional differences between embryonic and postnatal fat-associated tissues; Figure S5: Region-specific functional enrichment of genes differentially expressed between embryonic and postnatal fat-associated tissues (1); Figure S6: Region-specific functional enrichment of genes differentially expressed between embryonic and postnatal fat-associated tissues (2); Figure S7: PCA of bulk RNA-seq samples by stage; Figure S8: Sensitivity analysis of postnatal abdominal region-associated transcriptional signatures and direct comparison between AR and VR; Figure S9: Combined and stage-split UMAP visualization of annotated single-nucleus RNA-seq cell populations from the abdominal region; Table S1: Sample metadata and bulk RNA-seq sequencing and alignment statistics; Table S2: Differentially expressed genes between embryonic and postnatal fat-associated tissues; Table S3: Functional enrichment of genes differentially expressed between embryonic and postnatal fat-associated tissues; Table S4: Associations between global WGCNA modules and developmental stages; Table S5: Functional enrichment of embryonic stage-associated WGCNA module group M1; Table S6: Functional enrichment of postnatal stage-associated WGCNA module group M2; Table S7: Associations between embryonic WGCNA modules and anatomical regions; Table S8: Functional enrichment of region-associated modules in the embryonic WGCNA network; Table S9: Associations between postnatal WGCNA modules and anatomical regions; Table S10: Functional enrichment of region-associated modules in the postnatal WGCNA network; Table S11: Quality-control summary of embryonic and postnatal single-nucleus RNA-seq datasets; Table S12: Cluster-enriched marker genes identified in the single-nucleus RNA-seq datasets; Table S13: Representative marker genes and literature references supporting single-nucleus cell-type annotations; Table S14: Differentially expressed genes between embryonic abdominal region and other fat-associated tissues; Table S15: Differentially expressed genes between postnatal abdominal region and other fat-associated tissues; Table S16: Functional enrichment of abdominal region-associated differentially expressed genes at the embryonic and postnatal stages; Table S17: Functional enrichment of embryonic abdominal region-associated genes overlapping with highly expressed preadipocyte and adipocyte genes; Table S18: Functional enrichment of postnatal abdominal region-associated genes overlapping with highly expressed preadipocyte and adipocyte genes; Table S19: Functional enrichment of adipose-lineage-enriched WGCNA modules negatively associated with the abdominal region; Figure S10: Expression patterns of top candidate hub genes in adipose-lineage marker-enriched WGCNA modules.

## Abbreviations

The following abbreviations are used in this manuscript:

2w	2 weeks post-hatch
E16	Embryonic day 16
AR	abdominal region
VR	visceral fat region
RNA	Ribonucleic acid
RNA-seq	RNA sequencing
snRNA-seq	Single-nucleus RNA sequencing
UMI	Unique molecular identifier
PCA	Principal component analysis
UMAP	Uniform Manifold Approximation and Projection
VST	Variance-stabilizing transformation
WGCNA	Weighted gene co-expression network analysis
DEG	Differentially expressed gene
FDR	False discovery rate
GO	Gene Ontology
BP	Biological process
CUBIC	Clear, Unobstructed Brain/Body Imaging Cocktails and Computational analysis
