# Supplementary material for: Integrated Bulk and Single-Nucleus Transcriptomics Characterizes the Developmental and Regional Specialization of Chicken Abdominal Fat-Associated Tissue
Source: Animals (Basel). 2026 Sep 14;16(18):2893. doi: 10.3390/ani16182893 (PMC13603698; doi:10.3390/ani16182893)
Supplement: Supplementary file 1 [file animals-16-02893-s001.zip › animals-4508610-supplementary/Supplementary Figure.pdf]

Integrated Bulk and Single-Nucleus Transcriptomics Characterizes the Developmental and Regional Specialization of Chicken Abdominal Fat-Associated Tissue

Siyu Zhang [2,e], Qihong Zhang [3,e], Jiali Wang [1] and Zhenhui Li [1,*]

[1] State Key Laboratory of Swine and Poultry Breeding Industry, Guangdong Provincial Key Lab of Agro-Animal Genomics and Molecular Breeding, College of Animal Science, South China Agricultural University, Guangzhou, Guangdong, 510642, China
[2] Fujian Key Laboratory of Animal Genetics and Breeding, Institute of Animal Husbandry and Veterinary Medicine, Fujian Academy of Agricultural Sciences, Fuzhou, Fujian, 350013, China
[3] State Key Laboratory of Swine and Poultry Breeding Industry, and Guangdong Laboratory for Lingnan Modern Agriculture, Guangzhou, Guangdong, 510642, China
* Correspondence: lizhenhui@scau.edu.cn

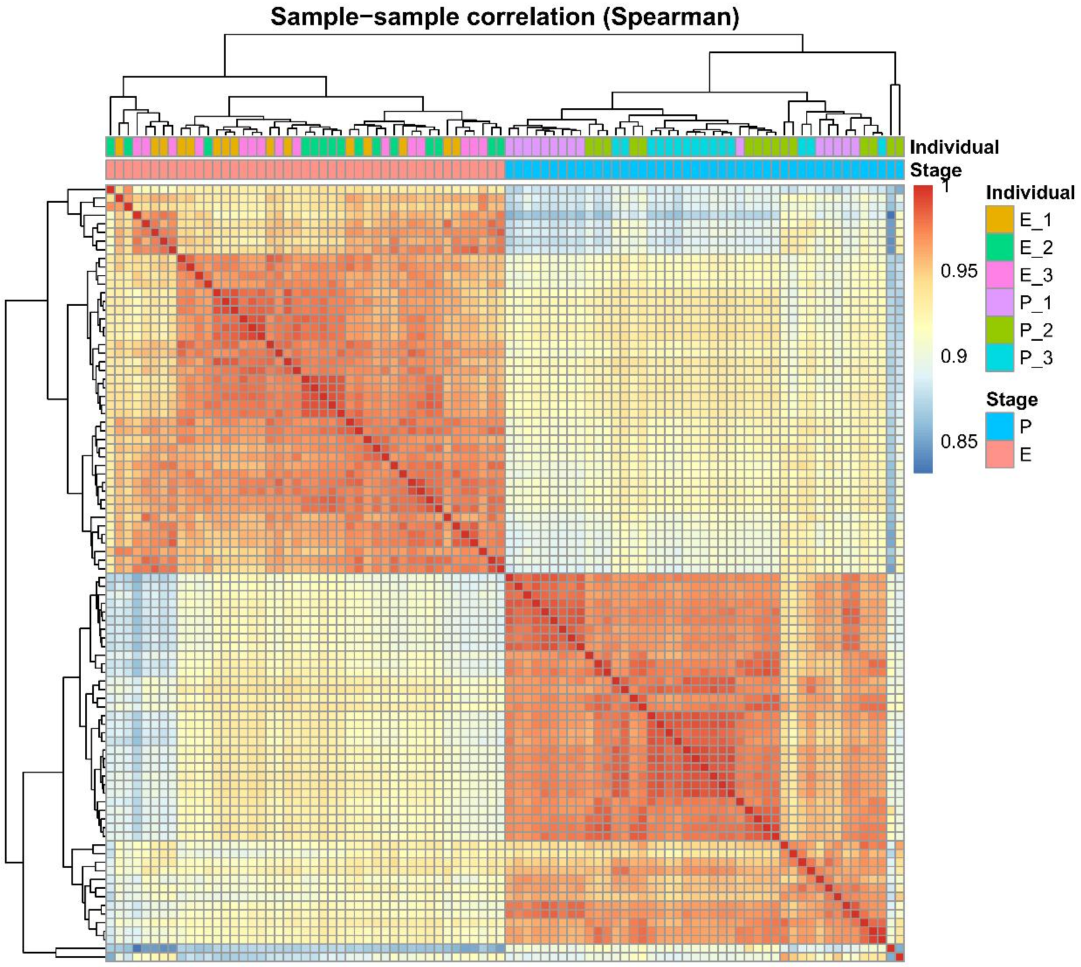

Figure S1. Sample–sample correlation structure of the 93 bulk RNA-seq tissue transcriptomes.

Spearman correlation coefficients were calculated between all pairs of samples using variance-stabilized expression values. Rows and columns were hierarchically clustered based on correlation-derived distances. Annotation bars indicate developmental stage, anatomical region, and biological individual. The heatmap was used to assess the overall similarity of transcriptomic profiles and the consistency of biological replicates across anatomical regions and developmental stages.

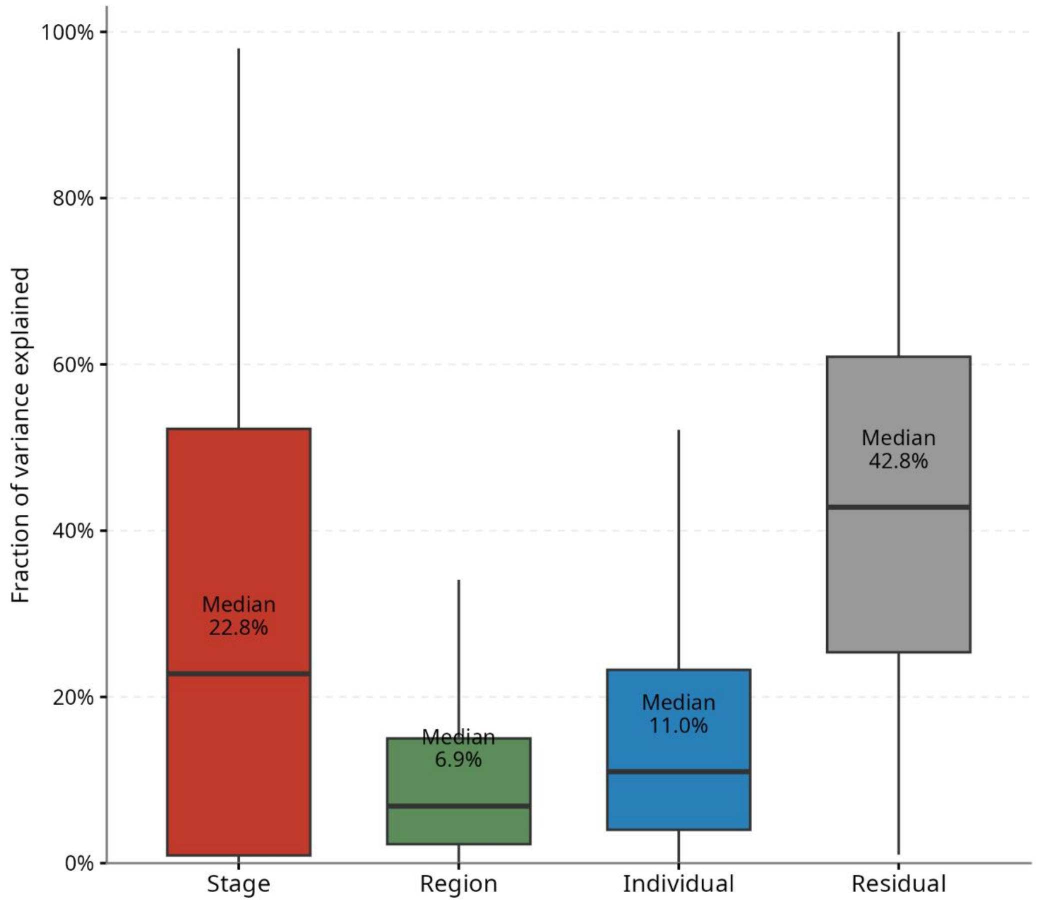

Figure S2. Gene-wise variance partitioning of bulk RNA-seq expression across developmental stage, anatomical region, biological individual, and residual variation. Variance fractions were estimated for each gene using a variance-partitioning model including developmental stage, anatomical region, and individual identity as random effects. Boxplots show the distribution of the fraction of expression variance attributable to each component across genes, with median values indicated above the corresponding distributions. Variance partitioning was used descriptively to characterize major sources of expression variation and was not used for developmental-stage hypothesis testing.

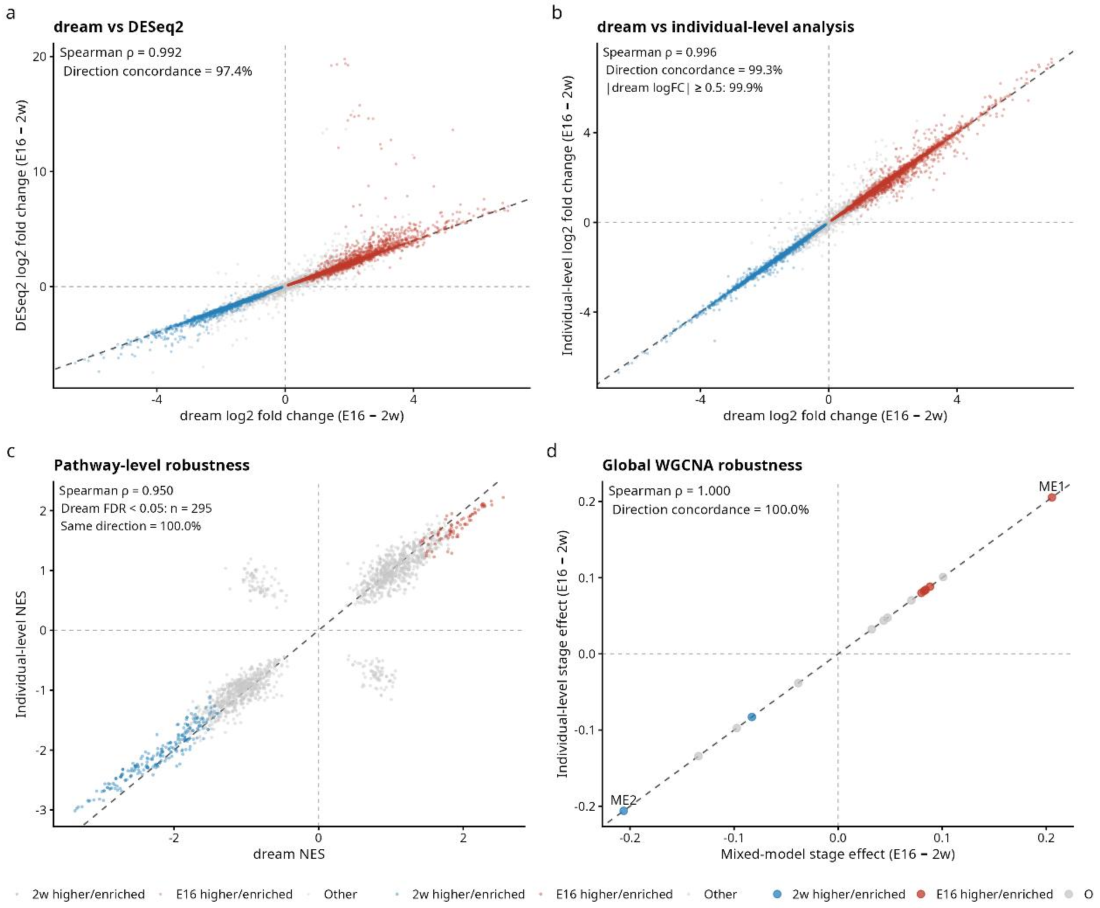

Figure S3. Robustness of the global embryonic-to-postnatal transcriptomic comparison across alternative analytical frameworks.

(a) Comparison of gene-level log2 fold-change estimates between the repeated-measures-aware dream mixed-effects model and the DESeq2 fixed-effects analysis using the same 90 tissue transcriptomes from the 15 anatomical regions shared between E16 and 2w. (b) Comparison between dream log2 fold-change estimates and those obtained from the individual-level sensitivity analysis, in which expression values were equally averaged across the 15 shared regions within each of the six biological individuals. (c) Comparison of normalized enrichment scores (NES) from GO Biological Process GSEA between the dream analysis and the individual-level sensitivity analysis. Positive NES values indicate enrichment at E16, whereas negative values indicate enrichment at 2w. (d) Comparison of developmental-stage effects on global WGCNA module eigengenes estimated using the repeated-measures mixed model and the six-individual sensitivity analysis. Positive effect estimates indicate higher eigengene expression at E16, whereas negative estimates indicate higher expression at 2w. Spearman correlation coefficients and directional concordance are shown in the corresponding panels.

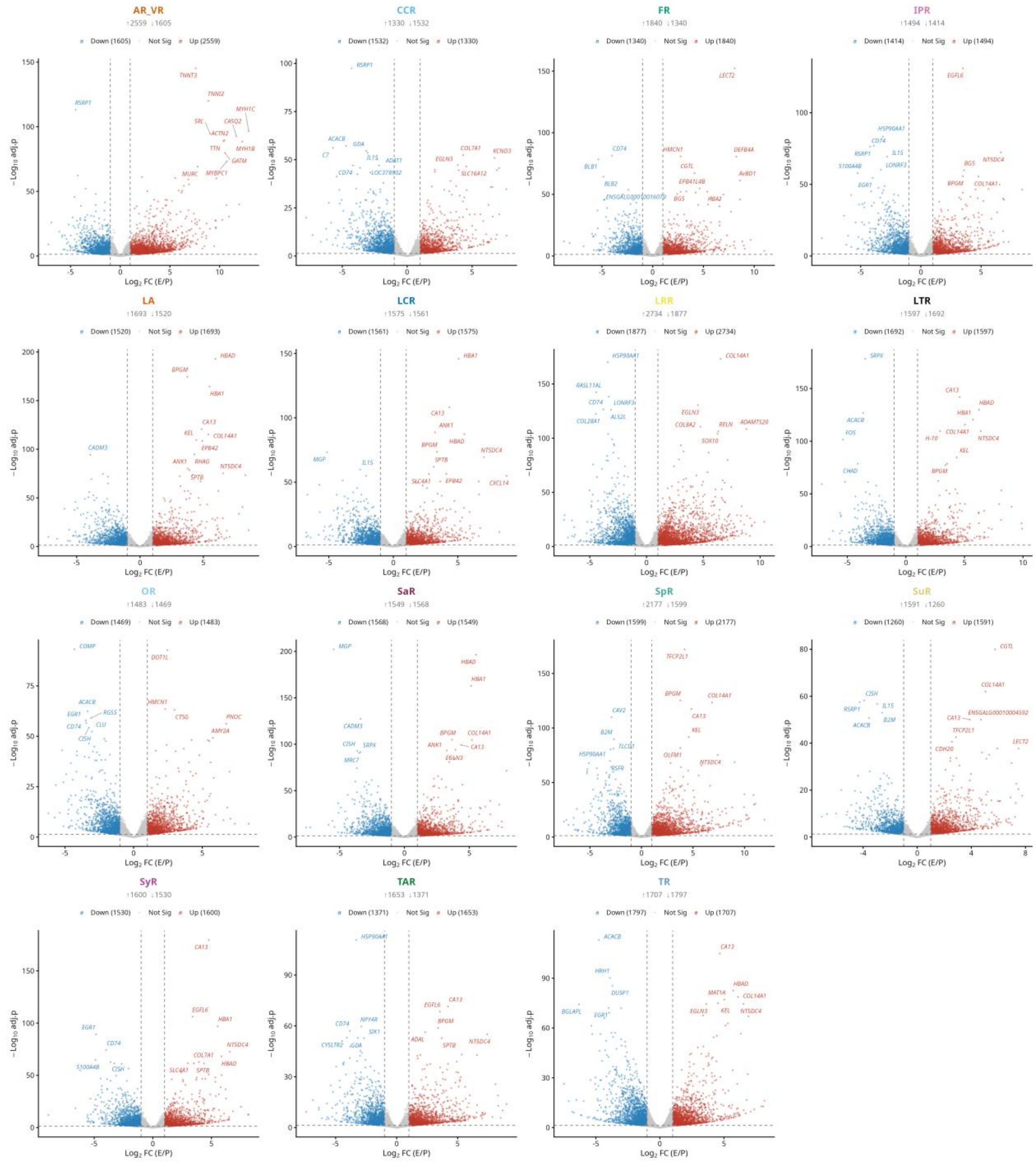

Figure S4. Region-specific transcriptional differences between embryonic and postnatal fat-associated tissues.

Volcano plots show differentially expressed genes between embryonic day 16 (E16) and 2 weeks post-hatch (2w) within each anatomical region. Each point represents one gene, with the log2 fold change on the x-axis and the -log10-adjusted P value on the y-axis. Genes with significantly higher expression at E16 and 2w are shown in red and blue, respectively, whereas nonsignificant genes are shown in grey. Differentially expressed genes were defined by an adjusted P value < 0.05 and an absolute log2 fold change > 1. The anatomical regions were AR, abdominal region corresponding to abdominal fat; CCR, clavicular-coracoid region; FR, femoral region; IPR, ischiopubic region; LA, lateral abdominal region; LCR, lateral cervical region; LRR, leg root region; LTR, lateral thoracic region; OR, ophthalmic region; SaR, sartorius region; SpR, spinal region; SuR, subalar region; SyR, synsacral region; TAR, transverse abdominal region; and TR, tracheal region.

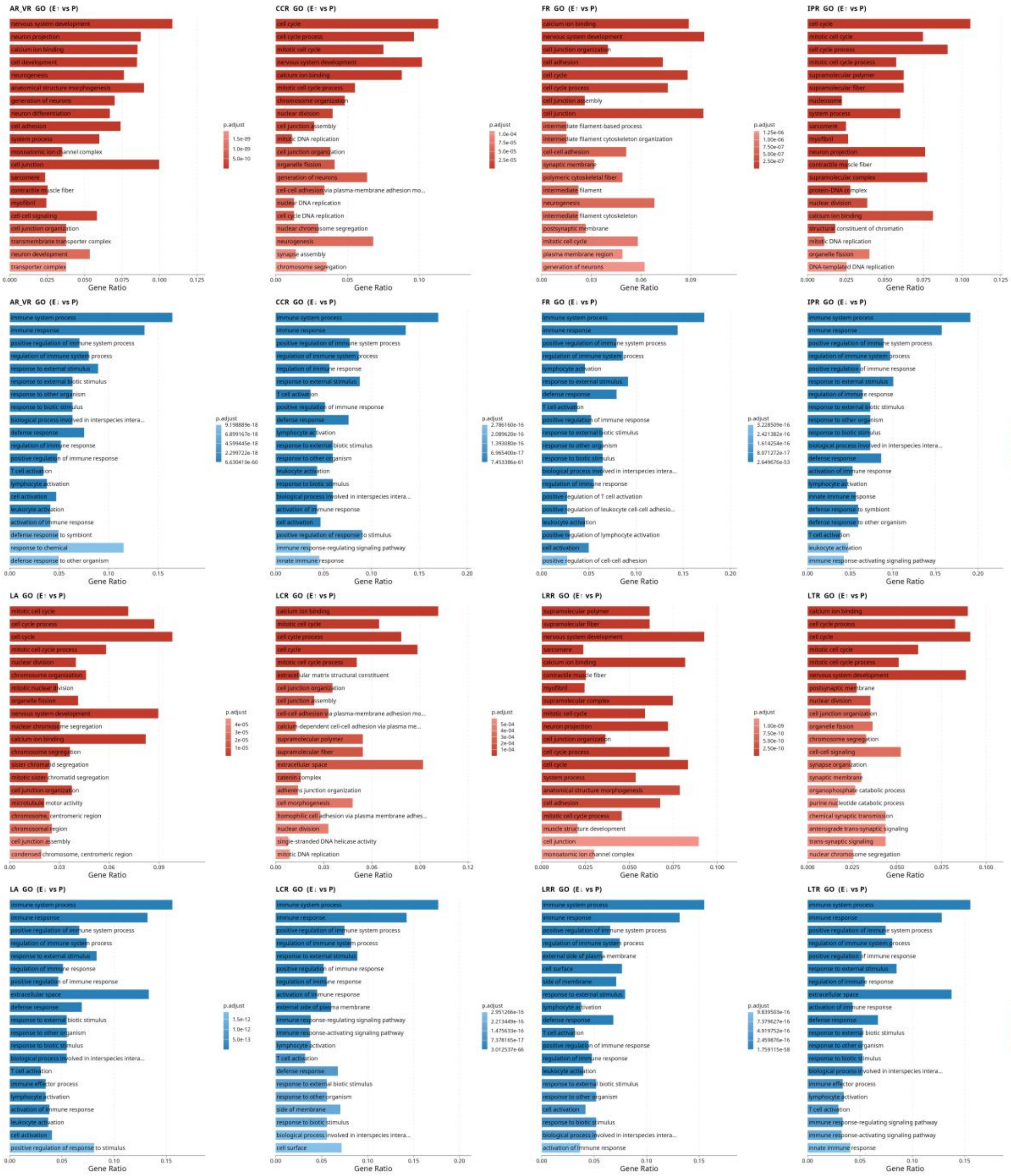

Figure S5. Region-specific functional enrichment of genes differentially expressed between embryonic and postnatal fat-associated tissues (1).

Gene Ontology biological process enrichment analysis was performed separately for genes showing higher expression at embryonic day 16 (E16) and 2 weeks post-hatch (2w) within each anatomical region. Red bars indicate biological processes enriched among genes upregulated at E16 relative to 2w, whereas blue bars indicate processes enriched among genes upregulated at 2w relative to E16. Bar length represents the gene ratio, and color intensity represents the Benjamini-Hochberg-adjusted *P* value. AR, abdominal region corresponding to abdominal fat; CCR, clavicular-coracoid region; FR, femoral region; IPR, ischiopubic region; LA, lateral abdominal region; LCR, lateral cervical region; LRR, leg root region; and LTR, lateral thoracic region.

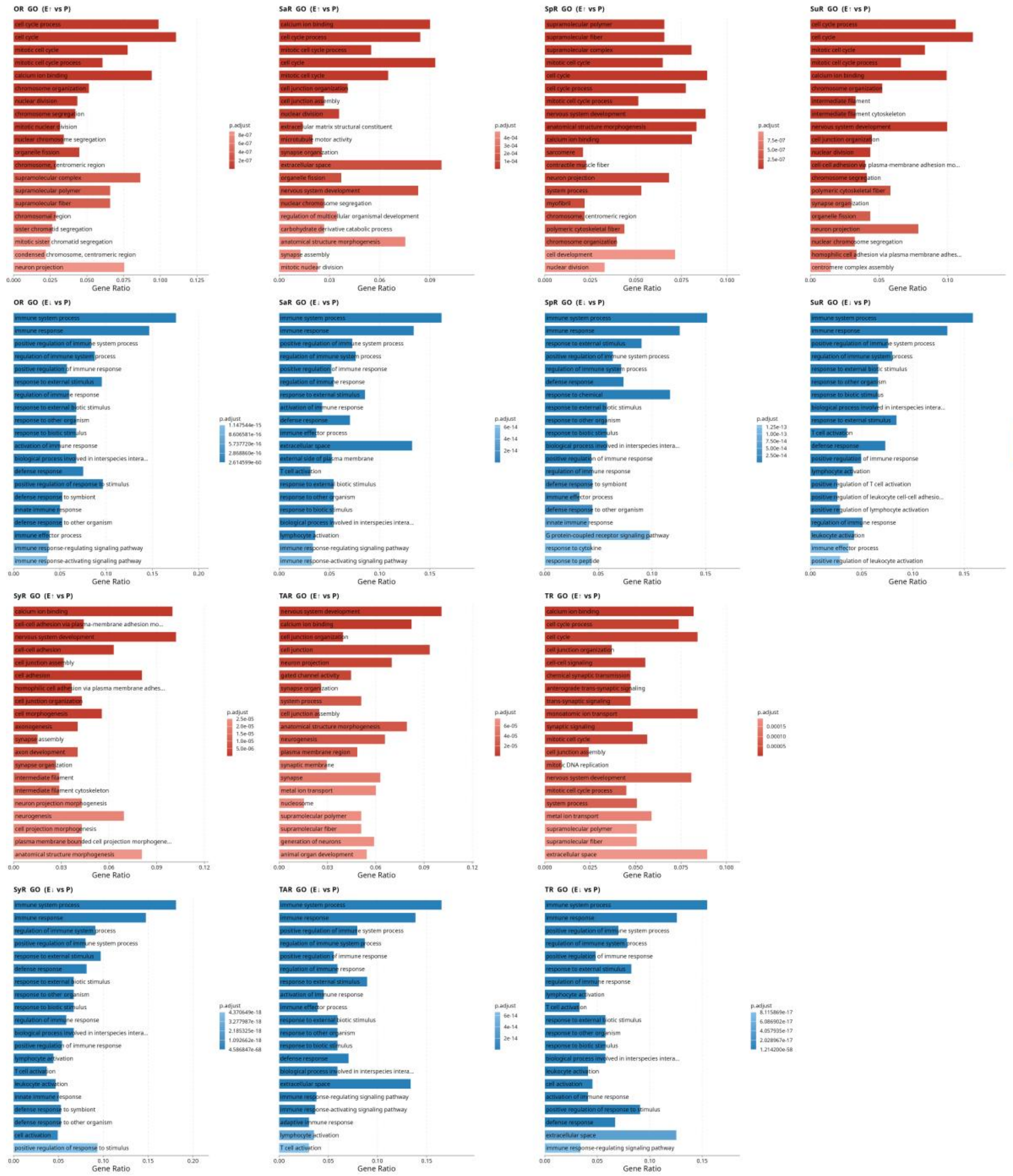

Figure S6. Region-specific functional enrichment of genes differentially expressed between embryonic and postnatal fat-associated tissues (2).

Gene Ontology biological process enrichment analysis was performed separately for genes showing higher expression at embryonic day 16 (E16) and 2 weeks post-hatch (2w) within each anatomical region. Red bars indicate biological processes enriched among genes upregulated at E16 relative to 2w, whereas blue bars indicate processes enriched among genes upregulated at 2w relative to E16. Bar length represents the gene ratio, and color intensity denotes the Benjamini–Hochberg-adjusted *P* value. OR, ophthalmic region; SaR, sartorius region; SpR, spinal region; SuR, subalar region; SyR, synsacral region; TAR, transverse abdominal region; and TR, tracheal region.

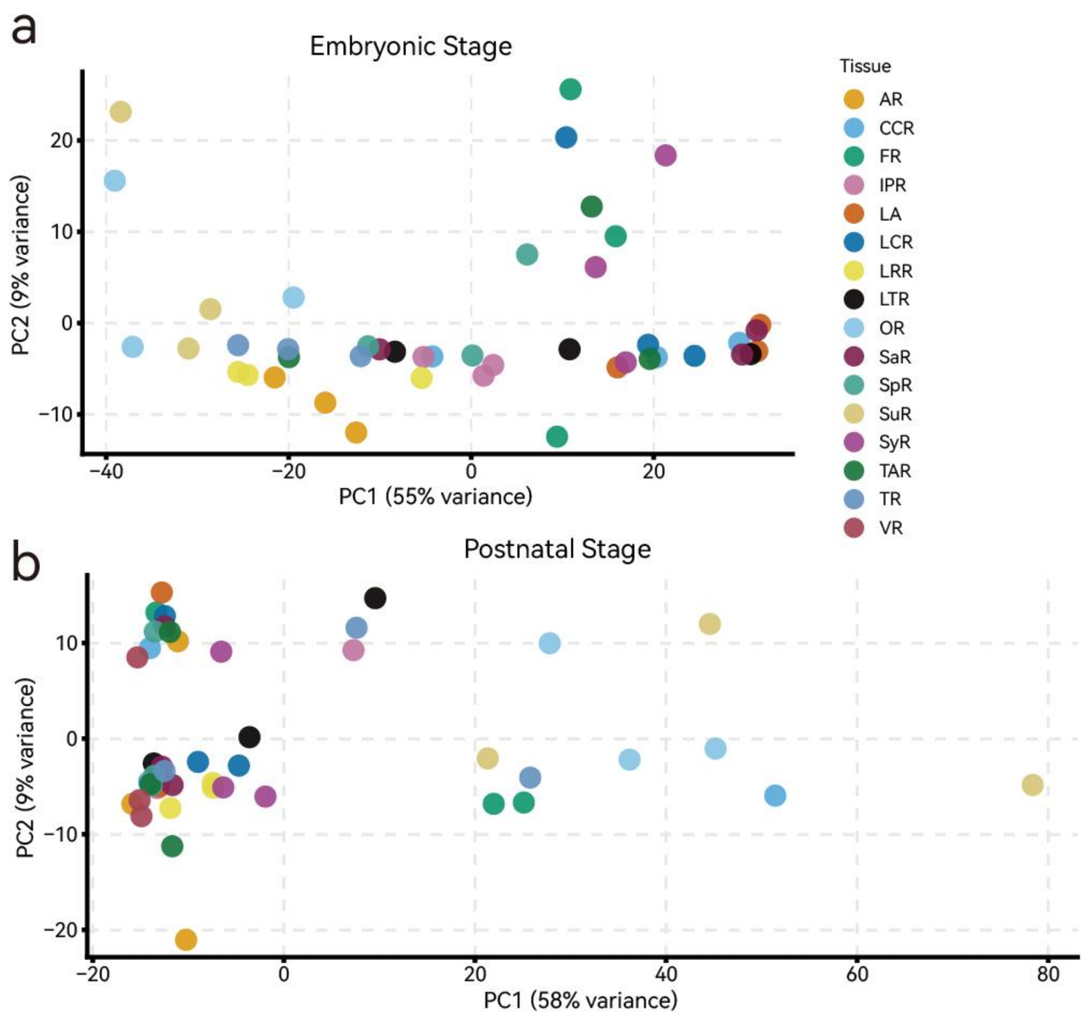

Figure S7. Stage-specific principal component analysis of bulk RNA-seq samples.
(a,b) PCA of embryonic (E16) and postnatal (2w) bulk RNA-seq samples analyzed separately. Each point represents one tissue transcriptome, and colors indicate anatomical regions. The percentages on the axes indicate the variance explained by the corresponding principal components.

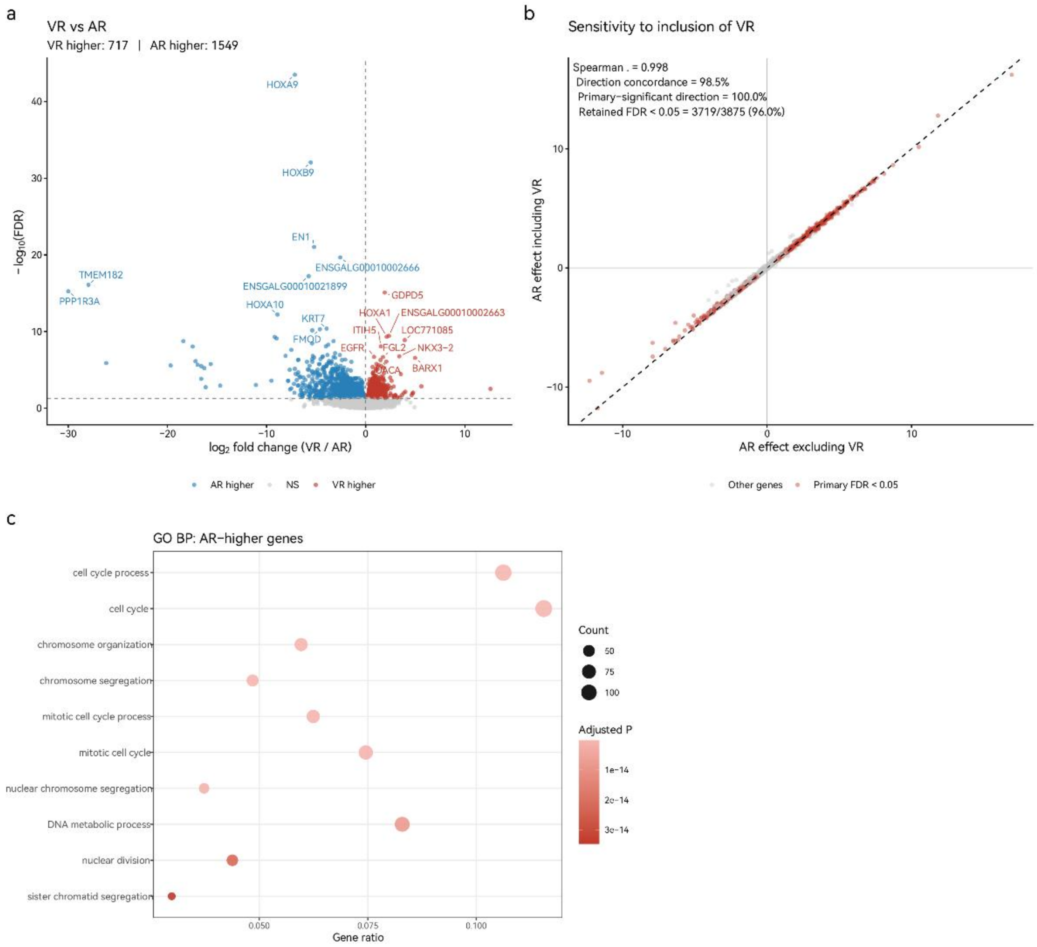

Figure S8. Sensitivity analysis of postnatal abdominal region-associated transcriptional signatures and direct comparison between AR and VR.

(a) Volcano plot showing differential gene expression between the postnatal visceral fat region (VR) and abdominal region (AR). Positive log2 fold-change values indicate higher expression in VR, whereas negative values indicate higher expression in AR. Genes with FDR < 0.05 are highlighted according to the direction of differential expression.

(b) Comparison of estimated AR effects obtained from the primary analysis excluding VR from the reference group and the sensitivity analysis including VR. The dashed line indicates identity. Spearman correlation, overall directional concordance, directional concordance among genes significant in the primary analysis, and the proportion of primary significant genes retaining FDR < 0.05 after inclusion of VR are shown.

(c) GO Biological Process enrichment of genes expressed at significantly higher levels in AR than in VR. Dot size represents the number of genes, the x-axis represents the gene ratio, and dot color represents the adjusted P value. No GO Biological Process terms reached statistical significance among genes expressed at higher levels in VR.

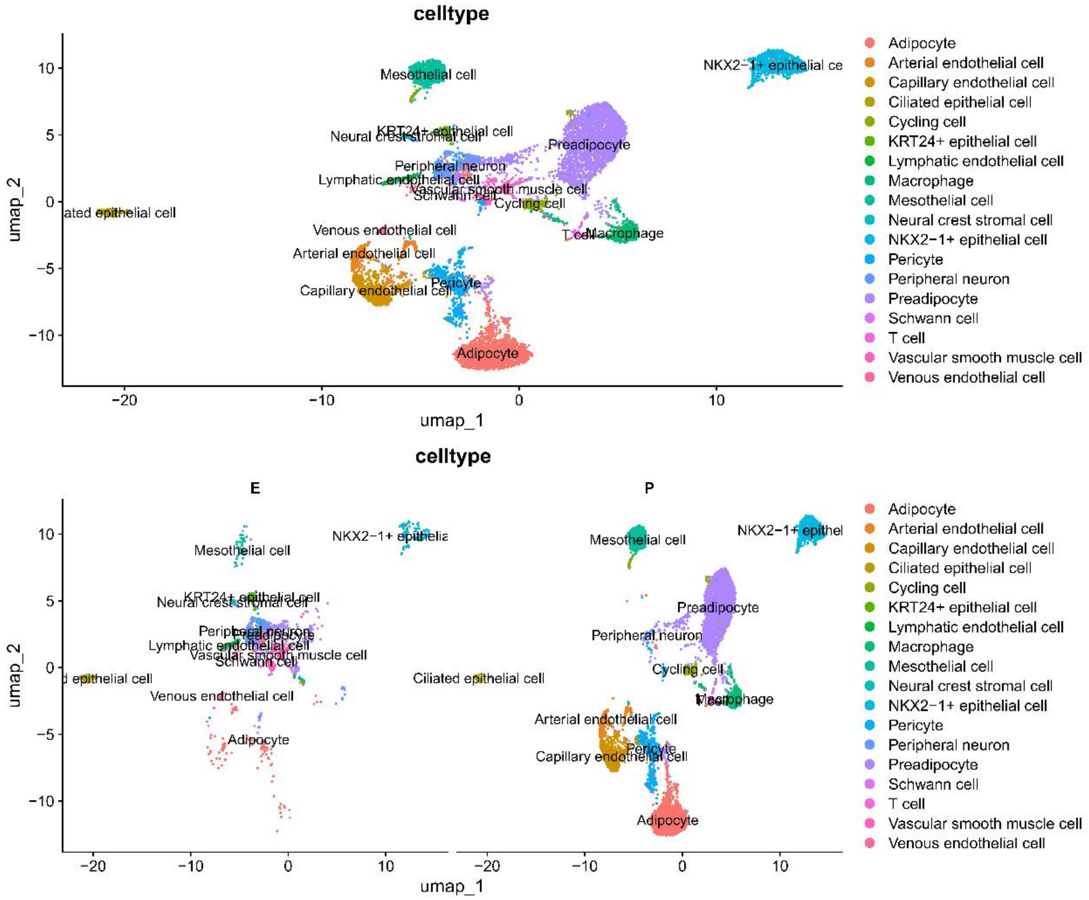

Figure S9. Combined and stage-split UMAP visualization of annotated single-nucleus RNA-seq cell populations from the abdominal region.

The upper panel shows the joint UMAP embedding of embryonic and postnatal nuclei after integration, colored by annotated cell type. The lower panels display the same embedding split by developmental stage (E, embryonic; P, postnatal), illustrating the distribution of annotated cell populations from each sample within the integrated embedding. These plots were used to support joint cell-type annotation and visualization and were not used to infer developmental differences in cell abundance because only one biological individual was analyzed per stage.

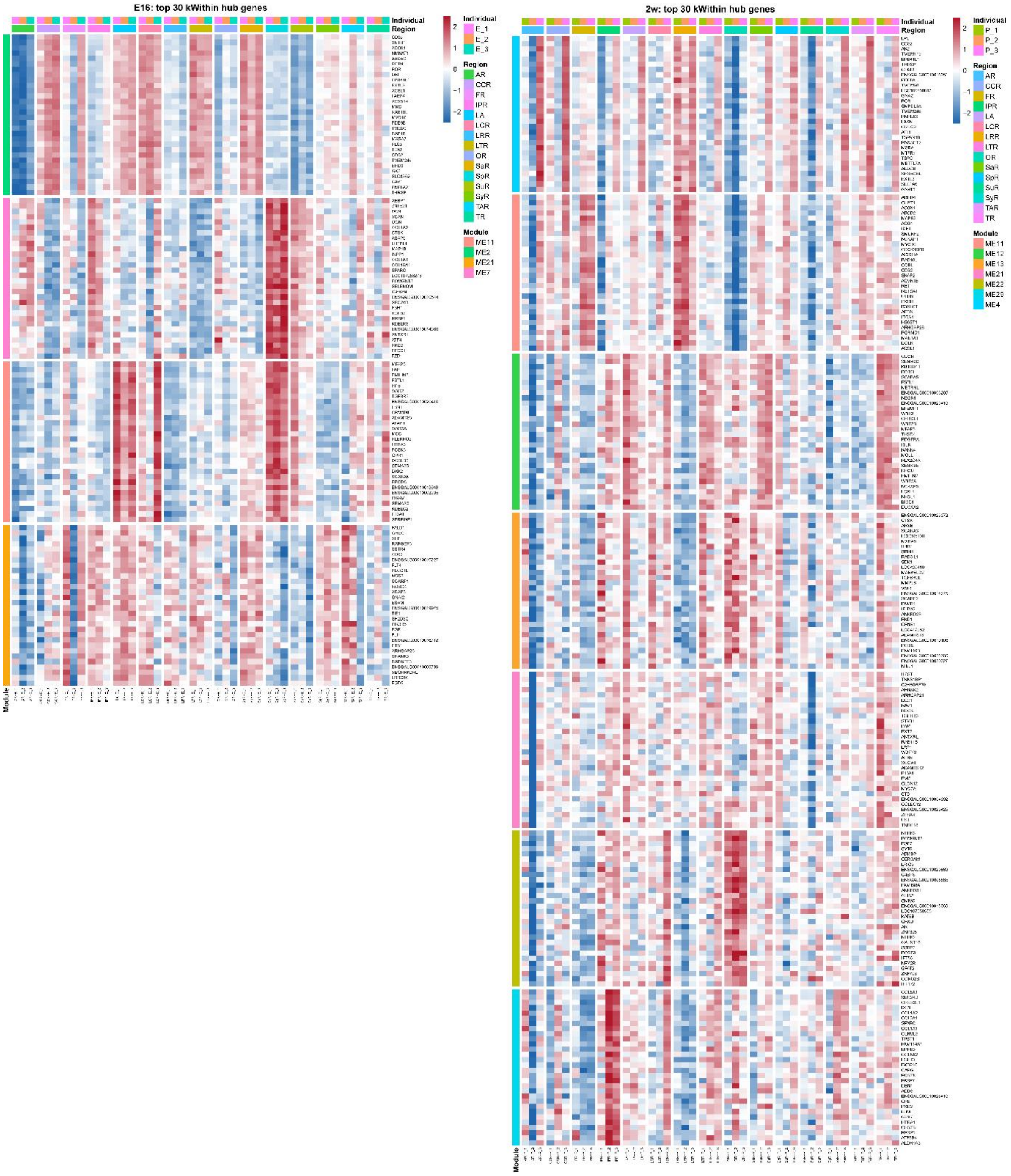

Figure S10. Expression patterns of top hub genes in adipose-lineage marker-enriched WGCNA modules. The left panel shows embryonic (E16) modules ME2, ME7, ME11, and ME21, whereas the right panel shows postnatal (2w) modules ME4, ME11, ME12, ME13, ME21, ME22, and ME29. For each module, the top 30 genes ranked by intramodular connectivity (kWithin) were selected. Expression values were standardized by gene across samples and are displayed as row-wise Z-scores. Samples are ordered by anatomical region and biological individual.
